# Neurobiological effects of gallic acid: current perspectives

**DOI:** 10.1186/s13020-023-00735-7

**Published:** 2023-03-15

**Authors:** Md. Shimul Bhuia, Md. Mizanur Rahaman, Tawhida Islam, Mehedi Hasan Bappi, Md. Iqbal Sikder, Kazi Nadim Hossain, Fatama Akter, Abdullah Al Shamsh Prottay, Md. Rokonuzzman, Eda Sönmez Gürer, Daniela Calina, Muhammad Torequl Islam, Javad Sharifi-Rad

**Affiliations:** 1 Department of Pharmacy, Bangabandhu Sheikh Mujibur Rahman Science and Technology University, Gopalganj, 8100 Bangladesh; 2 Department of Pharmacy, Southern University Bangladesh, Chattogram, 4210 Bangladesh; 3 Faculty of Pharmacy, Department of Pharmacognosy, Sivas Cumhuriyet University, Sivas, Turkey; 4 Department of Clinical Pharmacy, University of Medicine and Pharmacy of Craiova, 200349 Craiova, Romania; 5 Facultad de Medicina, Universidad del Azuay, Cuenca, Ecuador

**Keywords:** Gallic acid; neuroprotective activity; pharmacological effects, Molecular mechanisms, Brain health

## Abstract

Gallic acid (GA) is a phenolic molecule found naturally in a wide range of fruits as well as in medicinal plants. It has many health benefits due to its antioxidant properties. This study focused on finding out the neurobiological effects and mechanisms of GA using published data from reputed databases. For this, data were collected from various sources, such as PubMed/Medline, Science Direct, Scopus, Google Scholar, SpringerLink, and Web of Science. The findings suggest that GA can be used to manage several neurological diseases and disorders, such as Alzheimer’s disease, Parkinson’s disease, strokes, sedation, depression, psychosis, neuropathic pain, anxiety, and memory loss, as well as neuroinflammation. According to database reports and this current literature-based study, GA may be considered one of the potential lead compounds to treat neurological diseases and disorders. More preclinical and clinical studies are required to establish GA as a neuroprotective drug.

## Introduction

According to the Pan American Health Organization (PAHO), there were 32.9 fatalities per 100,000 population (age-standardized), 33.1 deaths per 100,000 population in males, and 32.2 deaths per 100,000 population in women due to neurological conditions in 2019 [1]. Considering the high mortality and morbidity rates in many developed and developing nations, many of these neurological disorders must respond unsatisfactorily to standard treatments. As conventional drugs are inadequate, neuroscientists are increasingly interested in developing new therapies based on traditional medicines with fewer side effects [57, 103]. Polyphenols are probably the most common type of compound found in natural resources [153]. Phenolic acids are phytochemicals called polyphenols that are often observed in plants as secondary metabolites (Rahaman et al.). To date, a significant range of biological activities have been identified for polyphenolic compounds [53, 90]. Gallic acid (GA), a type of phenolic acid, has been associated with a wide range of neurological ailments. It is a secondary metabolite found in many different parts of the upper plant kingdom in free-state or ester form [61]. It is a colorless to slightly yellow crystalline substance and is used in the food and medicine industries [52]. Oils and fats can be prevented from oxidizing and becoming rancid by using GA and its derivatives, such as lauryl and propyl gallate. GA and some of its derivatives can be used as preservatives and dietary ingredients, directly or indirectly, for human consumption. It can be used in cosmetics because it protects cells from radiation [32]. Aside from the previously mentioned application, several in vitro and in vivo neuropharmacological activities have been reported for GA, such as Alzheimer’s disease [175], Parkinson’s disease [134], anxiety [110], depression [140], epilepsy [65], neuropathic pain [82], sedation [105], cerebral ischemia [164], and psychosis [185]. It has also been revealed to be anti-inflammatory [178] and effective against cancer [186], gastrointestinal diseases [14], and cardiovascular diseases [79]. The purpose of this study is to summarize the neurological effects of GA and its derivatives based on database reports.

## Methodology

A search was done in the following databases: PubMed/Medline, Science Direct, Scopus, Google Scholar, SpringerLink, Web of Science, and numerous patent offices (as-USPTO, CIPO, WIPO) using the next MeSH terms: “Gallic Acid/pharmacology”, “Gallic Acid/administration & dosage”, “Cerebral Cortex/drug effects”, “Cerebral Cortex/pathology”, “Hippocampus/drug effects”, “Neuroprotective Agents/pharmacology”, “Disease Models, Animal”, “Alzheimer Disease/drug therapy”, “Parkinson Disease/psychology”, “Brain Ischemia/drug therapy”, “Plant Extracts/administration & dosage”, “Anti-Anxiety Agents/pharmacology”, “Antidepressive Agents/administration & dosage”. The articles were evaluated for information about the neuropharmacological activity of GA, concentration or dose, route of administration, test procedures either in vivo or in vitro, results or possible mechanisms of action, and a final summary, as well as the underlying reasons for various neurodegenerative diseases. The taxonomy of the plant has been validated according to the World Flora Online and the chemical structures according to PubChem [127, 180]. The most representative data have been included in tables and figures.

### Inclusion criteria

1. Studies carried out *in vitro*, in silico, *ex vivo*, or *in vivo* with or without utilizing laboratory animals, including mice, rats, rabbits, and humans, and their derived tissues or cells.
2. Studies with GA or its derivatives or preparations.
3. GA or its derivatives provide joint activity with other chemical compounds.
4. Studies with or without suggesting possible mechanisms of action.

### Exclusion criteria

1. Studies demonstrated data duplication and titles and/or abstracts not meeting the inclusion criteria.
2. GA with other studies uncovering the current issue.
3. Papers written in other languages than English.

## Phytochemistry and natural sources of gallic acid

**Fig. 1:**
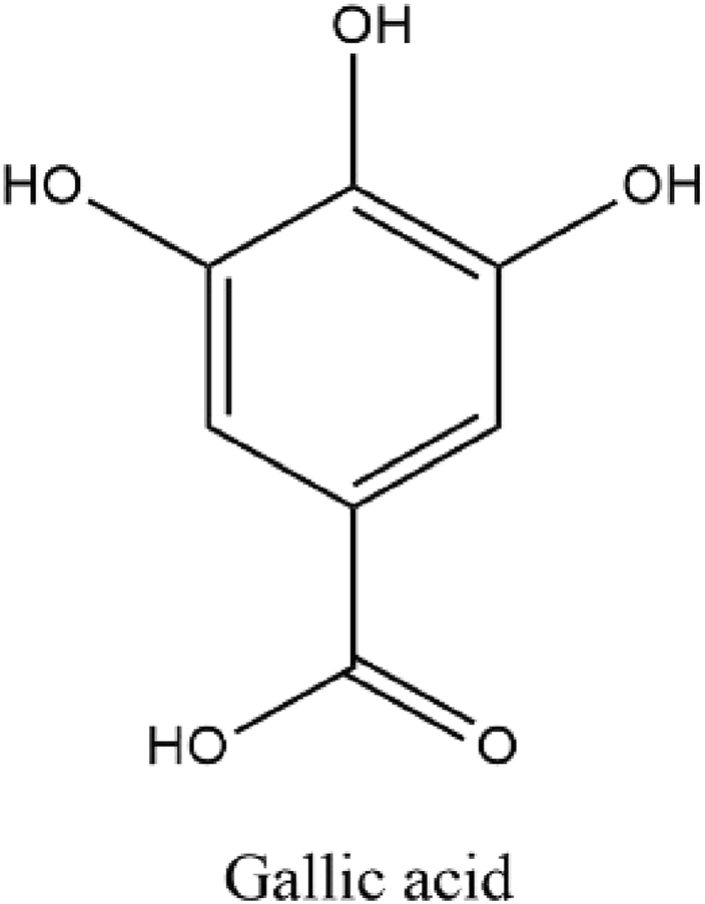
Chemical structure of gallic acid (3,4,5-trihydroxy benzoic acid)

GA, also known as 3,4,5-trihydroxy benzoic acid (Fig. 1), an organic acid having phenol and carboxylic acid properties, has only one benzene ring structure. The chemical formula of GA is C_7_H_6_O_5_ with a MW of 170.12 g. It is either a colorless or slightly yellow crystalline powder with melting temperatures ranging from 235 to 240 °C (it decomposes). When heated to 100–120 °C, it loses crystal water due to structural instability [126]. GA is generated chemically through the hydrolysis of tannic acid with the aid of sulfuric acid at temperatures between 110 and 120 °C [92].

There are various natural sources of GA, such as strawberries, blueberries, tea, blackberries, grapes, mangoes, walnuts, cashew nuts, hazelnuts, wine, plums, and other foods that contain GA [38]. It is also found in many plants, including bearberry leaves, pomegranate root bark, sumac, witch hazel, oak bark, tea leaves, and many more, both free and as part of the tannin molecule [11]. Some plant species that have GA include the bark of *Quercus robur*, the root of *Pueraria lobata*, the fruits of *Guazuma ulmifolia*, the fruits of S*ambucus nigra*, the fruit and peels of *Syzygium malaccense*, the leaves of *Sorocea guilleminina*, the stem and bark of *Abutilon pannosum*, the leaves and stem of *Barringtonia racemosa*, the seeds of *Camellia japonica*, fruits of *Antidesma bunius*, and so on [12].

## Bioavailability and pharmacokinetics of gallic acid

Drug discovery relies on pharmacokinetics (PK) to aid in the optimization of lead compounds’ absorption, distribution, metabolism, and excretion (ADME) properties, with the result being a clinical candidate with an appropriate concentration–time profile in the body to achieve the desired therapeutic effect without unacceptable adverse effects [24, 135, 171]. In clinical practice, medications are used if they have desirable pharmacokinetic features; for example, peptide drugs are becoming more widely used because of their efficacy [139, 170, 184]. Most therapeutic compounds in clinical development have problems with bioavailability because of their low solubility and absorption [35, 169].

Although GA is insoluble in chloroform, benzene, and petroleum ether, it dissolves in ether, glycerol, alcohol, and acetone in addition to water. Approximately 70 percent of GA is absorbed after oral treatment, with the remainder eliminated as 4-OMeGA in the urine. Reports indicate that treatment with *Polygonum capitatum* extracts at a dose of 60 mg/kg resulted in the highest GA content in the kidneys of rats, followed by the lungs, which demonstrated the second-highest level of GA, with only trace amounts found in the spleen, heart, and liver. No GA was detected in the brain tissue. Additionally, of the metabolite 4-OMeGA, approximately 16.67% of the ingested GA was eliminated unchanged in urine samples. Moreover, GA is converted into several derivatives upon digestion, thereby hindering its pharmacological efficacy due to widespread metabolism and clearance [113].

Using the HPLC method, Shahrzad et al. [146] determined the PK of tea and *Acidum gallicum* tablets (each occupying 0.3 mM of GA) in healthy individuals. The result of this study demonstrated that GA from both the tea and tablets was expeditiously absorbed and excreted with mean half-lives of 1.06 ± 0.06 and 1.19 ± 0.07 h and mean maximum concentrations of 2.09 ± 0.22 and 1.83 ± 0.16 μM/L (plasma), respectively. After administration of the black tea and tablets orally, 39.6 ± 5.1 and 36.4 ± 4.5% of the GA dose were excreted in urine as its metabolite (4OMGA) and GA, respectively. Where more than 60% of GA excreted was converted to 4OMGA [146]. Another study reported that alteration of the PK process in GA may occur in a normal or pathogenic condition. For example, oral administration of GA monohydrate at doses of 50 and 100 mg/kg to MI (myocardial infraction) rats exhibited slower absorption into the bloodstream than normal rats. Additionally, remarkable prolonged t_1/2_ and MRT, as well as diminished CL, were also registered in MI rats. This investigation suggests that MI can alter the PK procedure of GA [188].

Many studies have shown that GA is safe and effective, but its pharmacokinetic features (such as low absorption, poor bioavailability, and rapid elimination) severely restrict its use [48, 189]. The use of nanoformulation can improve these poor PKs [123]. It has been shown that using nanotechnology-based techniques improves the pharmacokinetic aspects of drugs [155]. Nanocarriers can increase the drug’s bioavailability and lipophilicity, which in turn improves the drug’s therapeutic effect (Javad SHARIFI-RAD 2022) The stability profile of a drug was another target of nanocarriers; they were also designed to improve encapsulation efficiency and allow for more precise control over drug release, all of which are essential for treating a variety of diseases [129, 130]. This means that nanotechnology has the potential to improve the therapeutic efficacy of medicine, leading to the desired pharmacological response that helps treat or cure humans. Practically distinct nanoformulation approaches applied to GA improved its pharmacokinetic characteristics. In a study, for instance, nanoformulation of the GA–phospholipid complex improved its bioavailability and hepatoprotective efficacy [16]. According to another study, elastic niosomes are particularly effective in enhancing the stability and permeability of GA for topical anti-aging use [102]. Managing neurodegenerative diseases (NDs) is complicated by the fact that drugs typically cannot enter the CNS without first penetrating the blood–brain barrier (BBB) [22, 46]. Nanomaterials pass across the BBB through both invasive and non-invasive processes. The BBB is ruptured using invasive physical methods, and nanomaterials are delivered across it via paracellular pathways such as intracerebroventricular or intracerebral injection, i.e., intranasal delivery strategy, receptor-mediated BBB crossing technique, cell-mediated BBB crossing strategy, shuttle peptide-mediated BBB crossing strategy, and cell-penetrating peptide (CPP). However, the BBB’s fundamental structure is maintained and not compromised by non-invasive drug delivery methods [183]. Because of advances in molecular-level monitoring, control, construction, repair, and diagnosis, nanotechnology, and more especially nanomedicine or pharmaceutical nanotechnology, offers a superior drug delivery strategy for NDs management [151] Nanoformulations of natural substrates are an efficient strategy for overcoming such obstacles and increasing the bioavailability of medications like GA [133].

## Neuropharmacological activities of gallic acid: underlying molecular mechanisms

### Effects on neurodegenerative diseases

#### Alzheimer’s disease

Alzheimer’s disease (AD) is an advancing neurological condition, which implies the symptoms develop over time and get progressively worse, causing the brain to atrophy and brain cells to die [70]. AD is the most common type of dementia and is associated with a cognitive decline drastic enough to interfere with everyday activities [160]. The disease is associated with the presence of abnormal neuritic plaques and neurofibrillary tangles. Plaques are spherical, microscopic defects with an extracellular amyloid beta-peptide (Aβ) layer that is generated by axonal terminal enlargement. An abnormal state of beta-amyloid 42 causes amyloid to aggregate, which promotes neuronal damage and loss of forebrain cholinergic neurons and commonly tends to dementia [144, 179]. Current treatment procedures are insufficient for adequately avoiding AD symptoms [72]. The current therapeutic paradigm for AD combines pharmaceutical and nonpharmacological methods to reduce increased cognitive and functional decline [9]. Brain-derived neurotrophic factor (BDNF) performs a regulatory function in synaptic plasticity, neural differentiation, and cell death procedures. Multiple regions of the brain, especially the hippocampus, have been found to contain BDNF [117], and there is a correlation between low BDNF levels and AD [88]. A higher level of BDNF increases brain performance. Trimethyltin (TMT) chloride is a toxin that contributes to the progression of AD and is frequently liable for BDNF level decreases [15]. However, tumor necrosis factor-alpha (TNF-α) plays a remarkable function in the CNS's response to damage [15, 158]. The leading risk factor for AD is the rise in TNF-α in persons with mild cognitive deprivation [74]. GA at the doses of 50 and 100 mg/kg in rats increased the hippocampal level of BDNF more than the TMT toxic rats as well as the hippocampal level of TNF-α to benefit AD patients [15]. GA at the dose of 30 mg/kg also increased passive avoidance and memory performance as well as enhanced non-enzymatic or enzymatic functions such as dismutase (SOD), catalase (CAT), and glutathione peroxidase (GPx) activities with diminishing the level of thiobarbituric acid (TBARS) substance in the hippocampus areas in intracerebroventricular- streptozotocin (STZ) (ICV-STZ) induced AD rates [104].

**Fig. 2:**
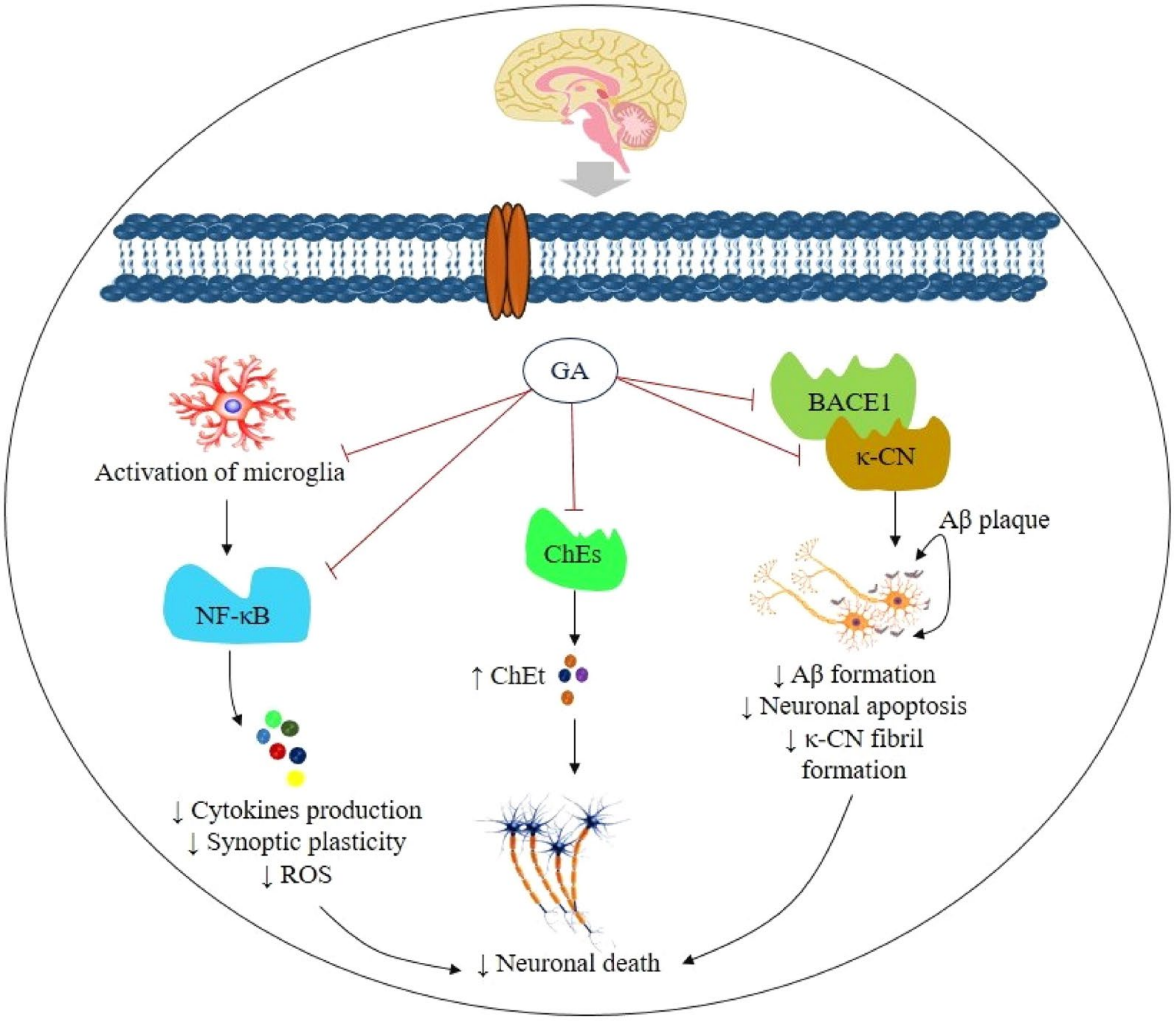
Schematic representation of the possible action pathways of gallic acid in Alzheimer’s disease. Abbreviations and symbols: ↑ increase, ↓decrease, *GA* gallic acid, *BACE1* beta secretase-1, *κ-CN* kappa-casein, *Aβ* amyloid beta, *ROS* reactive oxygen species, *ChEt* cholinergic transmitter, *ChEs* cholinesterase, *NF-κB* nuclear factor kappa-light-chain-enhancer of activated B cells

Possible mechanisms of action for GA in Alzheimer’s disease are shown in Fig. 2.

#### Parkinson’s disease

Parkinson's disease (PD) is a progressive neurodegenerative disorder marked by rigidity, tremor, and bradykinesia, with some patients acquiring postural instability as the disease progresses [40]. It is related mostly to the gradual loss of dopaminergic neurons (DPNs) in the substantia nigra of the brain [39, 43].

Involuntary mouth motions (IMMs) are significant symptoms of a variety of disorders or pharmacological situations, including PD and tardive dyskinesia, respectively [6, 39, 97]. GA prevents IMMs, such as in an in vivo experiment in which GA at the doses of 13.5 and 40.5 mg/kg diminished vacuous chewing movements (VCMs) in rats induced by reserpine at a dose of 1 mg/kg [134]; in another investigation, GA also reduced VCMs and catalepsy at the dose of 150 mg/kg, where VCMs and catalepsy were induced by tacrine and haloperidol at the doses of 2.5 mg/kg and 1 mg/kg, respectively, in rats [81]. Some investigations have demonstrated that administering reserpine to animals reduces the degree of certain antioxidant defenses and enhances oxidative indicators in rats [2, 166], and recent research has shown that several naturally occurring antioxidants can reduce involuntary spasm in reserpine-mediated animals [13, 21, 26, 134]. The effect of GA against VCMs and catalepsy is due to its antioxidant properties [81, 86].

**Fig. 3:**
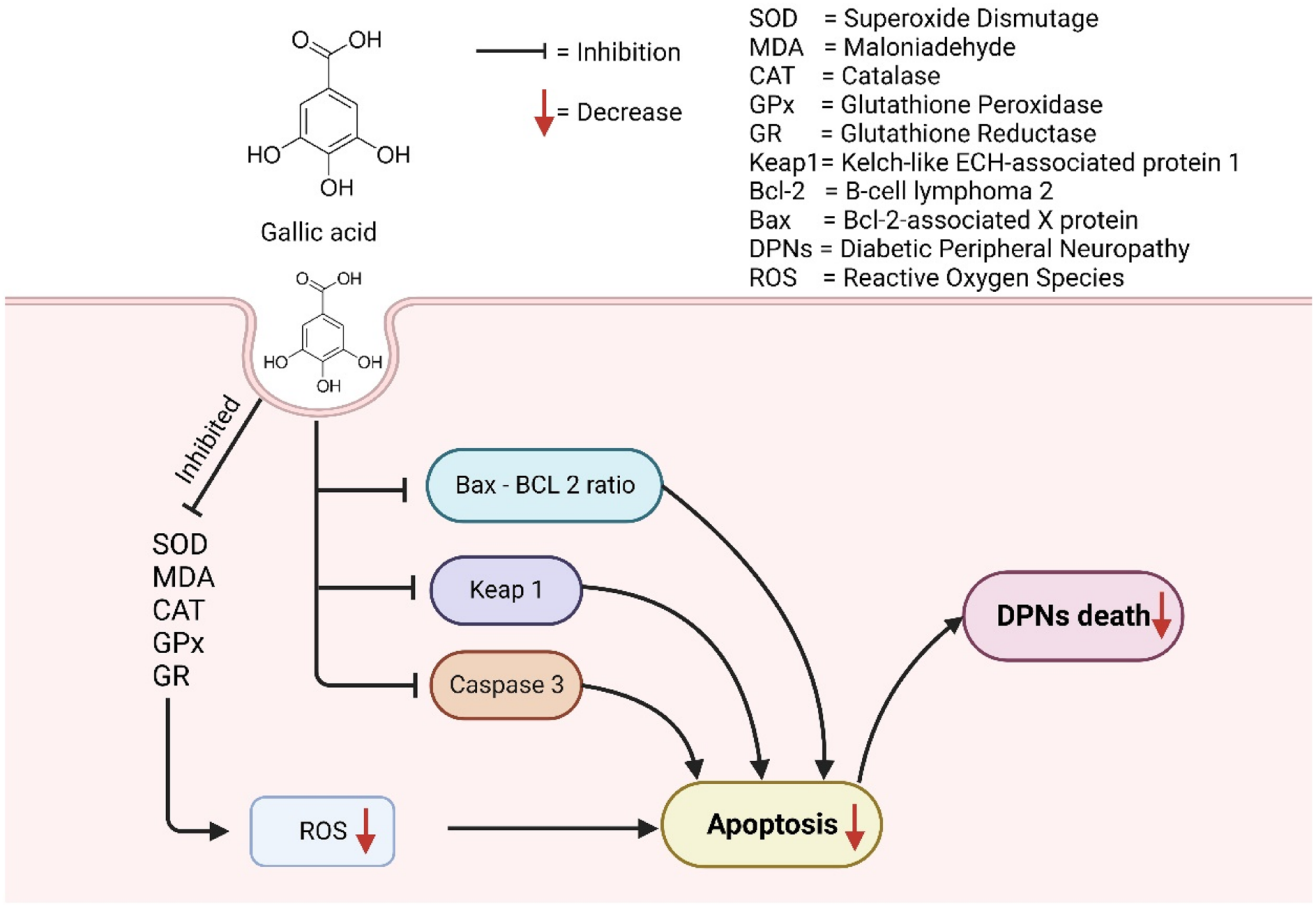
Possible anti-Parkinson mechanism of gallic acid

6-Hydroxydopamine (6-OHDA) induces apoptosis in human DPNs, SH-SY5Y [168]. The 6-OHDA promotes the generation of free radicals such as O2•, OH•, and NO•, resulting in nigrostriatal DPgc lesions [69]. By provoking multiple signaling cascades, including Nrf2, Keap-1, PI (3)K, caspases, MAPKs, and p53, excessive ROS generation may cause damage to DPNs [76, 112]. Pretreatment by GA at the concentration of 0.25–2.5 μg/ml reverts the up-regulation of Keap-1 and caspase-3 and, down-regulation of Nrf2, BDNF and p-CREB as well as also diminished the ratio of Bax and Bcl-2 proteins resulting protection of DPNs benefiting PD patients [27]. In another in vivo investigation, GA at the doses of 50, 100, and 200 mg/kg prevents memory deficit and cerebral oxidative stress mediated by 6-OHDA injected in the medial forebrain bundle in rates of the PD model. The antioxidant feature of GA is mostly responsible for its anti-PD action, which enhances the total thiol, and GPx, as well as diminishes malondialdehyde (MDA) and TBARS levels [103]. The possible anti-Parkinson mechanism of GA is shown in Fig. 3.

### Effects on psychiatric disorders

#### Anxiety

Anxiety is an involuntary neurophysiological condition that plays a fundamental role in negative emotions [8, 37]. It is believed that norepinephrine, serotonin (5-HT), dopamine (DP), and gamma-aminobutyric acid (GABA) are the mediators of anxiety in the CNS [31, 136]. Additionally, anxiety and chronic stress are significantly associated with memory issues, and the risk of AD and other kinds of dementia owing to chronic stress reduces the volume of the hippocampus (HIP), a brain area that is severely affected in individuals with memory loss [54, 100].

**Fig. 4:**
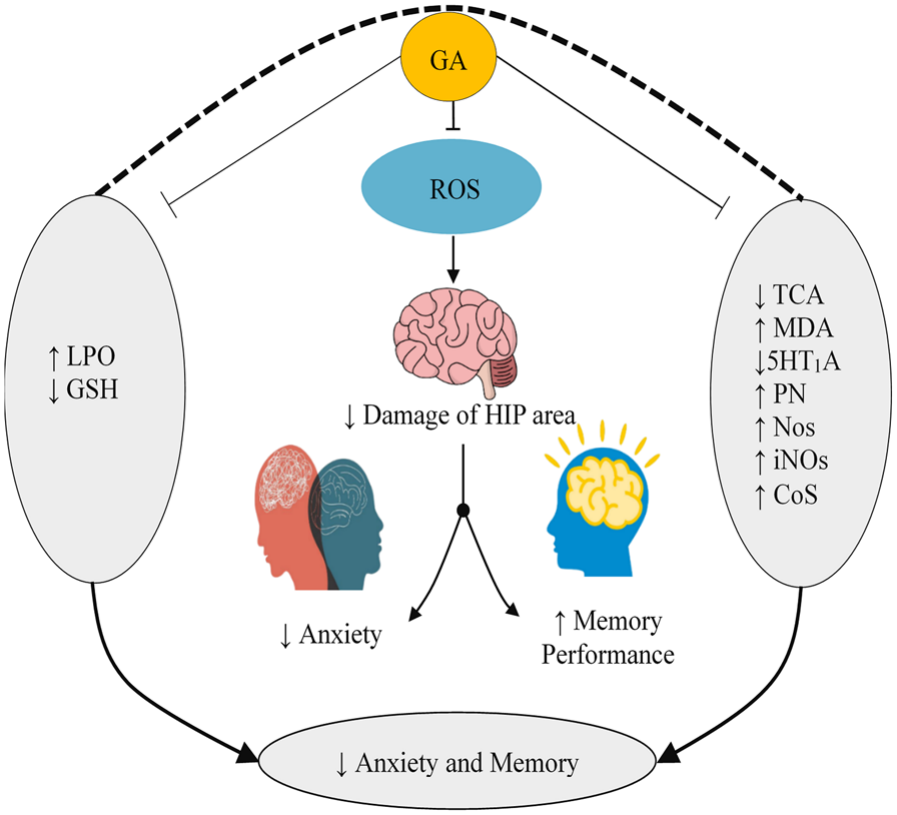
Anxiolytic and memory enhancing pathways of gallic acid. ↑ increase, ↓decrease, *GA* gallic acid, *ROS* reactive oxygen species, *TCA* tricarboxylic acid, *MDA* malondialdehyde, *5HT*_*1A*_ 5-hydroxytryptamine (serotonin) receptor 1A, *PN* plasma nitrite, *Nos* nitric oxide synthase, *CoS* corticosterone, *iNOs* Inducible nitric oxide synthase, *LPO* lipid peroxidation, *GSH* glutathione, HIP hippocampus

Recent studies reported that GA can generate anxiolytic-like activity in STZ-induced diabetes rats by elevating lipid peroxidation (LPO) levels and enhancing the reduction of glutathione (GSH) in the HIP and PFC (prefrontal cortex) [124]. GA can also diminish anxiety by increasing brain TCA and lowering elevated levels of serum and brain MDA in mice at doses of 5, 10, and 20 mg/kg b.w. [137]. An investigation by Mansouri et al. [105], reported that GA at lower doses (20 or 300 mg/kg) demonstrated a 5-HT_1_A receptor agonistic effect, resulting in an increase in the time spent and entries in the open arms of the elevated plus maze (EPM) test, which characterizes the anxiolytic activity [105]. GA-Nps and GA at the dose of 10 mg/kg in Swiss mice diminished plasma nitrite (PN) levels [110]. And in another investigation, GA at the doses of 5, 10, and 20 mg/kg administered to stressed and unstressed albino mice produced significant anxiolytic activity by inhibiting neuronal nitric oxide synthase in unstressed mice and by inhibiting iNOS and lowering plasma corticosterone levels in stressed mice [42]. Possible anxiolytic and memory-enhancing pathways of GA are shown in Fig. 4.

#### Depression

**Fig. 5:**
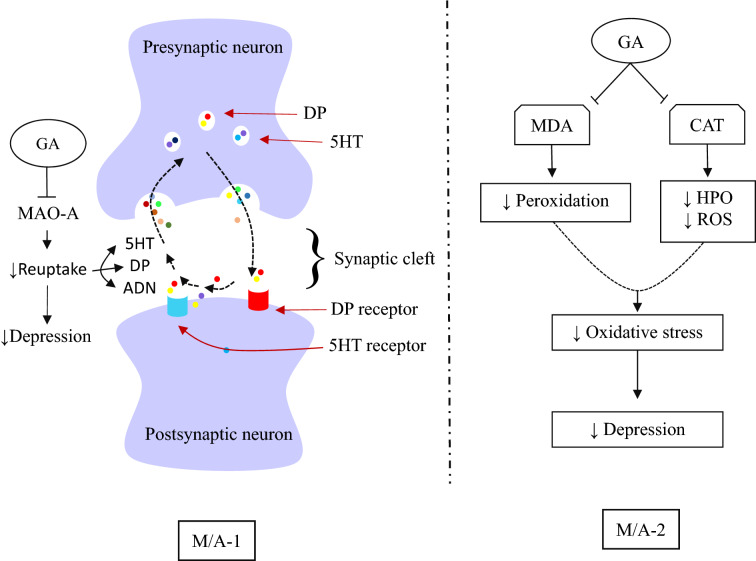
Gallic acid against depression: possible molecular interaction. ↑ increase; ↓decrease, *GA* gallic acid, *DP* dopamine, *5HT* serotonin, *ADN* adrenaline, *MAO-A* Monoamine oxidase A, *MDA* malondialdehyde, *CAT* catalase, *HPO* hydrogen peroxide, *ROS* reactive oxygen species, *M/A-1* MAO-A inhibition, *M-/A-2* antioxidant response

Depression is a potentially fatal condition that affects hundreds of millions of people and is primarily caused by environmental stressors such as immobility. Depression is believed to be triggered by an efficacious shortage of the monoaminergic neurotransmitters 5-HT, DP, or norepinephrine, while mania is characterized by an efficacious abundance of monoamines at key synapses [36, 143]. Monoaminergic systems are liable for numerous behavioral manifestations, including mood, alertness, motivation, and weariness, as well as psychomotor agitation or interruption. Unusual activity and behavioral repercussions of depression or mania may result from deflected neurotransmitter production, storage, or secretion as well as a receptor or intracellular messenger sensitivity [162]. Today, therapies with antidepressant drugs have become a formidable obstacle because of their low effectiveness rate and numerous unwanted effects. Therefore, it is of the utmost importance to discover better natural adjuvant therapies for these interrelated conditions [37].GA exhibited antidepressant-like activity in mice at the doses of 30 and 60 mg/kg b.w. by reducing immobility duration due to a binary M/A by enhancing not only 5HT but also DP or norepinephrine (catecholamine) levels in synaptic clefts of the CNS [25]. Post-stroke depression in mice was also inhibited at the doses of 25 and 50 mg/kg estimated through the reduction of immobility duration [108]. Antidepressant activity of GA in mice was also evaluated with the aid of the forced swim test (FST) and sucrose preference test by Chhillar and Dhingra [30], and the output of the study demonstrated that immobility in mice in the FST remarkably decreased due to the diminishing of monoamine oxidase-A (MAO-A) activity, MDA levels, and CAT function in unstressed mice and by preventing MAO-A activity, MDA, corticosterone, and PN levels in stressed mice [30]. In another investigation, GA nanoparticles at the dose of 10 mg/kg b.w. also ensured an antidepressant-like effect in mice by affecting immobility, MAO-A activity, MDA levels, as well as CAT function [111]. Figure 5 depicts possible anti-depressant pathways of GA.

#### Psychosis

Psychosis is one of the incapacitating psychiatric conditions characterized by a cluster of symptoms, including hallucinations, alogia, delusions, avolition, anhedonia, flat affect, and memory loss [47, 109, 185]. NMDA receptor antagonists modulate neurotransmitter systems in the CNS, including GABAergic, cholinergic, dopaminergic (DPgc), serotonergic, and glutamatergic systems, which are involved in psychosis [20, 122, 159]. NMDA receptors (NMDARs) hypofunction causes aberrant DPgc activity via increasing GABA release [89]. Psychosis is well reported to be significantly influenced by DPgc dysfunctions in the frontal cortex and limbic system [107].

GA reduced LP, restored total brain proteins and DP levels, and serum TNF-α and AChE activity enhanced the levels of GSH and GABA at the doses of 50, 100, and 200 mg/kg, p.o. in psychotic mice, where psychosis was induced through the administration of an NMDA receptor antagonist (ketamine) at the dose of 50 mg/kg [185].

### Effects on stroke

Stroke is a neurological condition that causes a significant loss of cerebral blood supply in a restricted region of the brain due to the abrupt or progressive occlusion of a major brain artery or the rupture of a brain blood artery [59, 66].

#### Ischemic stroke

Ischemic stroke**/**cerebral ischemia (CIS**)** is an acute cerebrovascular consequence linked with CNS tissue injury resulting from oxygen and glucose pauperism from a reduced or blocked brain artery [5, 29]; it involves cerebral hypoxia leading to ischemia neurons dying within a short time [23, 164]. Reperfusion may promote the anticipation of secondary brain injury because the newly incoming oxygen serves as a substrate for increased ROS creation [152, 164, 182]. Mitochondria played a key role in CIS injury with the aid of ROS production, mitochondrial malfunction, and type II apoptosis [34, 172]. In an in vitro investigation GA a the concentration of 0.1 or 1 μM reduced mitochondrial dysfunction, ROS production, and mitochondrial (type II) apoptosis resulting in reversed hypoxia/reoxygenation protection from cerebral ischemia and another in vivo investigation by the same investigator GA at the doses of 25, 37.5 and 50 mg/kg diminished infarct volume and the number of TUNEL (+) cells in MCAO rats prevents cerebral ischemia/reperfusion [164]. GA at dosages of 50, 100, and 200 mg/kg increases the antioxidant defense against BCCA occlusion-mediated ischemia/reperfusion in rats, indicating that it possesses neuroprotective properties [49].

#### Hemorrhagic stroke

Intracerebral hemorrhage develops within neural tissue or ventricles and is a leading CNS health issue with high morbidity and mortality rates worldwide [63, 91]. Microglia in the local area are activated (overactive) and liberate inflammatory cytokines and chemokines, aggravating brain tissue injury [3]. However, activated microglia can release TNF-α, IL, IFN, and TGF autocrine or paracrine [161]. Consequently, due to this secretion, inflammatory immune cells are also activated during an ischemia episode [181]. Moreover, these inflammatory cells subsequently exacerbate local tissue deterioration by increasing the development of free radicals, vasoactive amines, cytotoxic enzymes, and chemokines, which engage even additional immune cells at the location of damaged tissue [80]. GA administered at doses of 50, 100, and 150 mg/kg b.w. in C57BL/6 J mice reduced the level of M1 molecules (COX-2, iNOS, and MCP-1) and enhanced M2 molecules (CD206, Arg-1, and IL-10) of microglia, resulting in a reduction of brain edema, and increasing the integrity of the BBB to diminish ischemic brain injury [128].

### Effects on neuropathic pain

Neuropathy is an illness, not a symptom. It is caused by CNS and PNS deterioration and characterized by painful sensation and/or loss of sensation [10]. Paclitaxel (PT) is appropriate for numerous cancer conditions, but it induces neurodegeneration of peripheral nerve endings, causing agonizing neuropathy [33, 58, 150]. The fundamental harmful mechanism of PT alters microtubulin polymerization through the generation of ROS, BCL2 proteins and TNF-α; change of cellular pro and anti-oxidant enzymes; Ca^2+^ dyshomeostasis; and activation of mitochondrial permeability transition pores [45, 121, 163]. Multiple complex pathways make the management of neuropathic pain disease extremely hard, and existing conventional medications provide only symptomatic alleviation [82, 83]. GA demonstrated protection against neuropathic pain in mice at the doses of 20 and 40 mg/kg by decreasing total calcium, TBARS, TNF-α, MPO activity, superoxide anion, and GSH level where the pain induced through paclitaxel administration [82].

### Effects on brain tumors

Glioblastoma multiforme is the most prevalent malignant initial brain tumor in adults, arising from glial cells of the brain or spine [4, 41]. Normal brain tissue next to the tumor is invaded by diffuse glioma cells, which may render standard therapies such as surgery, radiation, and chemotherapy ineffective [17, 157]. The problem of drug resistance also hinders the efficacy of treatment for glioblastoma patients. Consequently, an efficacious therapy for glioblastoma must be established [167]. An in vitro investigation demonstrated that GA in both human glioma U251n and U87 cells inhibited glioma cell proliferation, viability, and invasiveness, as well as tube development in normal mouse brain endothelial cells, and it inhibited ADAM17 expression, which may be linked to the suppression of invasiveness through the deactivation of Ras/MAPK and PI3K/Akt signaling pathways [98].

Some studies have established the consequences of miRNAs on a distinctive set of physiological procedures, including cell development, proliferation, and apoptosis. Consequently, the deregulation of their expression is crucial for the beginning, development, and spread of malignant cells [7, 64, 154, 177]. In another in vitro investigation, GA at the higher dose of 100 µg/ml increased miR-17 levels, resulting in a reduction in mitochondrial antioxidant activity, slowed down T98G human glioblastoma proliferation, decreased the ability to repair the damage, and enhanced apoptosis [119]. GA also expressed cytotoxicity by influencing Ca^2+^ homeostasis and inducing Ca^2+^-linked cytotoxicity in DBTRG-05MG human glioblastoma cells, as well as subsequently, Ca^2+^ signal activating mitochondrial apoptotic pathways involving ROS generation [68].

### Sedation

Sedation is the process of calming down the body through a slowdown in brain activity [19]. Among various types of sedatives, a major part stimulates the activity of GABA receptors in the brain, producing a stronger sedation effect by lowering locomotor activity [114, 191]. GA in Wistar rats produces anxiolytic activity at a lower dose, but at a higher dose (500 mg/kg b.w.), it diminishes locomotor activity in the rats, resulting in sedation [105].

### Effects on neuroinflammation

Millions of people all around the world suffer from neuroinflammation after experiencing trauma or mental stress [149, 171]. Nearly every form of neurological disorder, including multiple sclerosis, stroke, AD, PD, and spinal cord injury, is a result of neuroinflammation [101]. The nigrostriatal dopaminergic system of the animal brain is affected by neuroinflammation as the disease progresses, as evidenced by glial cell activation, increases in proinflammatory cytokines (TNF-α, IL-1β, IL-6, and IFN-γ), and enzymes ((iNOS, COX-2), as well as protein aggregation, inflammasome activation, and cell death [94, 174, 176], Zhang and An, 2007). There have been many suggestions for neuroprotective techniques based on anti-inflammatory medications to restore damaged brain function [55, 77].

**Fig. 6:**
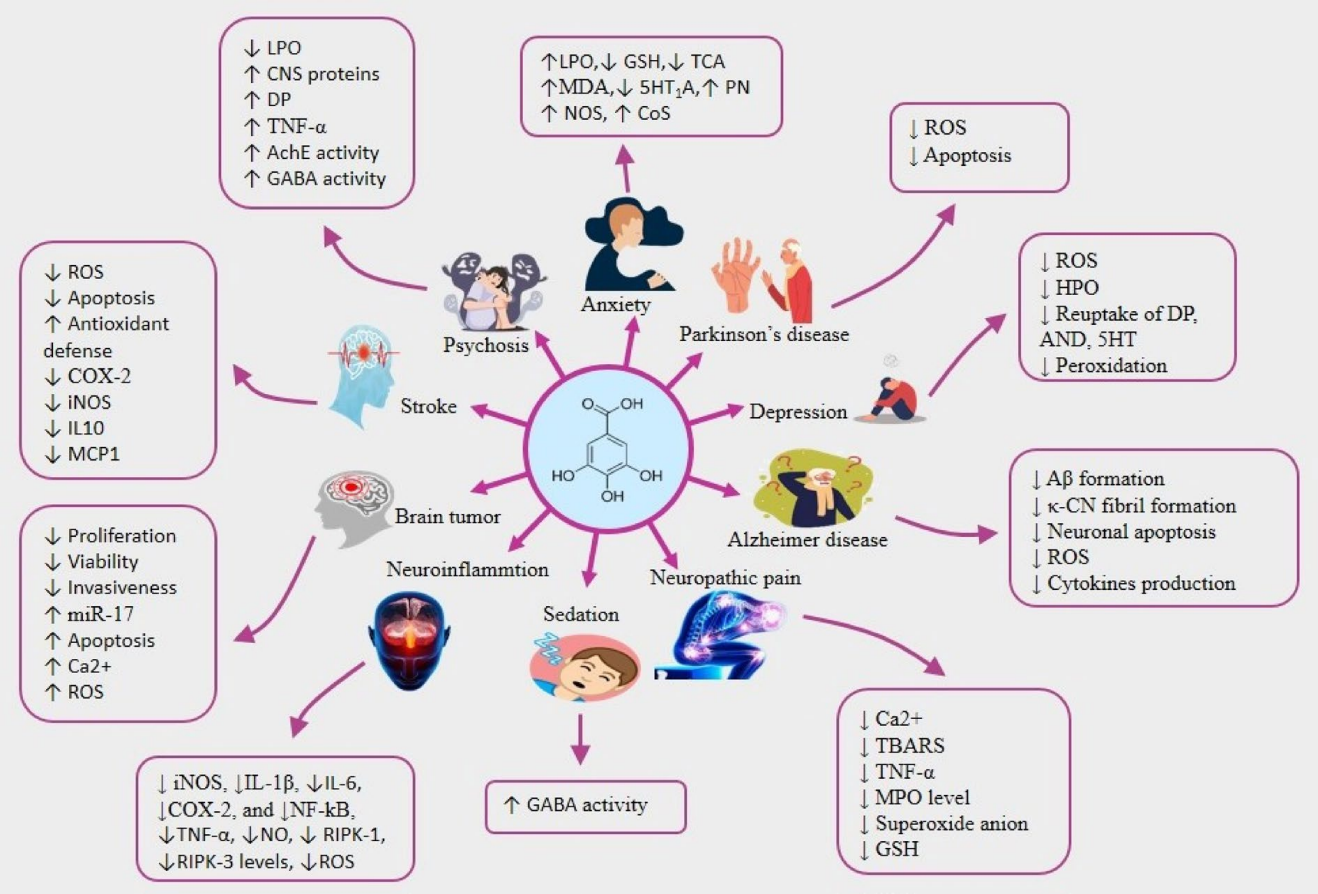
Overall neurological activities of gallic acid against different diseases and states of different mediators or proteins. ↑increase, ↓decrease. *Aβ* amyloid β protein, *TNF- α* tumour necrosis factor-α, *TBARS* 2-thiobarbituric acid reactive substances, *κ-CN* kappa-casein, *NF-kB* nuclear factor kappa B, *GSH* glutathione, *MDA* malondialdehyde, *TCA* tricarboxylic acid, *5-HT*_*1A*_ 5-hydroxytryptamine (serotonin) receptor 1A, *DP* dopamine, *AChE* acetylcholinesterase, *GABA* gamma-aminobutyric acid, *COX-2*: cyclooxygenase-2, *IL-6* interleukin-6, *IL-10* interleukin-10, *IL-1β* interleukin-1β, *ROS* reactive oxygen species, *MPO* myeloperoxidase, *RIPK-1* receptor-interacting protein kinase-1, *RIPK-3* receptor-interacting protein kinase-3,*NO* nitric oxide, *iNOS* inducible nitric oxide synthase, *LPO* lipid peroxidation, *MCP1* monocyte chemoattractant protein-1, *HPO* hydrogen peroxide, *AND* adrenaline; *PN* plasma nitrile, *NOS* nitric oxide synthase, *CoS* corticosterone

Various studies have reported that the phytochemical GA is a potential therapeutic for managing neuroinflammation [95]. A recent in vivo study exhibited that giving GA at a dose of 50–100 mg/kg decreased iNOS, IL-1β, heme oxygenase-1, RIPK-1, and RIPK-3 levels, α-synuclein aggregation, ROS production, and caspase 3 expressions in rats with neuroinflammation caused by LPS resulting reduction of inflammation [95]. In an in vitro study of BV2 microglial cells that had been treated with LPS, the same group of researchers found that GA could lower NO levels and iNOS expression at a concentration of 25–100 M [95]. A study by Siddiqui et al. [156] reported that GA had a strong anti-inflammatory effect at 1.0 µM by reducing the expression of COX-2, NF-κB, tenascin-C, and chondroitin sulfate proteoglycans in LPC-induced inflammatory hippocampal neurons co-cultured with glial cells [156]. The drug also decreased the production and expression of the glial fibrillary acidic protein in astrocytes, which led to the production of proinflammatory mediators and neurotoxic ROS that may start neuronal apoptosis, which can lead to AD and PD [75, 156]. Several studies (both in vivo and in vitro) make it strongly evident that the neuroinflammatory activity of GA is due to its capability of inhibiting inflammatory cytokines. For example, GA reduced the release and expression of TNF-α, IL-1β, IL-6, iNOS, COX-2, and NF-κB both in rats with traumatic brain injuries and oligomeric Aβ-mediated BV-2 and neuro-2A inflammatory cells resulting reduction of neuroinflammation [85, 142]. Table 1 and Fig. 6 displays the overall neuropharmacological activities of GA discovered in the literature.

**Table 1:** Overview of the effects of gallic acid in neurological diseases and disorders

Neurological disease	Experimental model	Concentration/Doses	Effects/Mechanisms	References
Alzheimer’s disease	Wistar rats (aluminium-chloride induced AD), in vivo	100 mg/kg b.w	↓CAT, ↓GSH, ↓SOD, ↓serum electrolyte, ↓neurotransmitter levels, ↑MDA, ↑ H_2_O_2_ and ↑NO	[115]
	*Drosophila melanogaster BL#33,798 cultures, *in vitro	IC_50_ = 50—100 µM	↓ Aβ, ↓ ChEs, ↓BACE-1	[116]
	APPswe/PS1dE9 transgenic mice (capable of Aβ plaque deposition at the age of 4 months), in vivo	30 mg/kg b.w	↓neurotoxicity, ↓ Aβ_1–42_	[187]
	Rats (AD induced through Aβ hippocampal injection), in vivo	50, 100, and 200 mg/kg b.w	↑cognitive function, ↓neural damage ↓Aβ plaques	[60]
	Rats (i.p. injection of trimethyltin, 8 mg/kg b.w.), in vivo	50 and 100 mg/kg b.w	↑ BDNF hippocampal level > TMT group > ↓ TNF-α hippocampal level	[15]
	Rats (intracerebroventricular–STZ injection), in vivo	30 mg/kg b.w	↑the passive avoidance, ↑memory ↑SOD, ↑GPx, ↑CAT, ↓TBARS	[104]
	Heochromocytoma 12 cells, in vitro	IC_50_ = 3.7 ± 0.3 µM	↓neurotoxicity ↓κ-CN activity, ↓Aβ peptide fibril	[96]
	Microglial, neuronal cells, in vitro	IC_50_ = 5–50 μM	↓ cytokines, ↓NF-kB, ↓neurotoxicity	[85]
	Mice, (Aβ _142_ and Aβ _421_ were administered by intracerebroventricular (ICV) injection), in vivo	10 and 30 mg/kg b.w	↓cognitive dysfunction, ↓ Aβ, ↓cytokines, ↓neuronal cell death	
Parkinson’s disease	Wistar male rats (catatonia induced by PPZ), in vivo	100, 200, 400 and 600 mg/kg b.w. (i.p)	↓catatonic responses	[67]
	SH-SY5Y cells, in vitro	IC_50_ = 0.25–2.5 μg/ml	↓neuronal cells damage, ↓ROS, ↓Keap-1, ↓caspase-3 ↓BDNF, ↓Nrf2, ↓p-CREB	[27]
	Rats received reserpine, in vivo	Dose = 13.5—40.5 mg/kg/day b.w	↓vacuous chewing movements	[134]
	Rats (PD induced through 6-OHDA; 8 μg/2 μL injected into the medial forebrain bundle), in vivo	Dose = 50, 100, and 200 mg/kg b.w	↑ memory ↑GPx, ↓ TBARS	[103]
	Rats (PD induced by apomorphine), in vivo	200 mg kg b.w	↓motor dysfunctions,↓ ROS and↓ gamma wave power,	[141]
	Rats, (tacrine 2.5 mg/kg b.w, i.p.), in vivo	150 mg/kg b.w	↓vacuous chewing movements	[81]
	Mice, (Haloperidol 1 mg/kg b.w, i.p.), in vivo		↓catalepsy	
Anxiety	Rats (HE mediated by bile duct ligation (BDL and NOR, open field and Morris water maze test), in vivo	20 and 30 mg/kg b.w	↑memory, ↑ AMPK pathway activity	[71]
	STZ-induced diabetic rats, in vivo	10, 20, and 40 mg/kg b.w	↓GSH in hippocampus and prefrontal cortex	[124]
	Mice acute and chronic stress in vivo	5, 10, and 20 mg/ kg b.w	↓serum and brain MDA levels ↑brain TCA	[137]
	Rats (EPM test), in vivo	30 and 300 mg/kg b.w	↑5-HT_1A_ receptor activity ↑time spent and entries in the open arms of elevated plus maze (EPM)	[105]
	Mice (EPM test), in vivo	GA nanoparticles: 10 mg/kg, 10 mg/kg b.w	↓plasma nitrite level	[110]
	Mice stress was generated by immobility, in vivo	5, 10, and 20 mg/kg b.w	↓plasma nitrite, ↓corticosterone levels	[42]
Depression	Rats anxiety- depression induced by sodium arsenite, in vivo	50 and 100 mg/kg b.w	↓immobility duration ↑time spent in open arm and light box	[140]
	Mice post-stroke depression, in vivo	30 and 60 mg/kg	↓immobility duration	[25]
	Mice (TST model), in vivo	25 and 50 mg/kg b.w	↓immobility duration	[108]
	Mice unpredictable chronic mild stress, in vivo	10 and 20 mg/kg b.w	↓immobility duration, ↓MDA, ↓MAO-A ↓plasma corticosterone levels	[30]
	Mice (DST and TST model), in vivo	10 mg/kg b.w. (GA nanoparticles)	↓immobility duration ↓ MDA, ↓MAO-A, ↓CAT	[111]
Psychosis	Mice (ketamine-induced psychosis), in vivo	50, 100, and 200 mg/kg, b.w. (p.o)	neuroprotective effects in psychosis ↓ LP, ↓DP,↓TNF-α, ↓AChE ↑ GABA, ↑glutathione	[185]
Sedation	Rats, in vivo	500 mg/kg (p.o.)	↓locomotor activity in rats	[105]
Strokes	C57BL/6 J mice, (MCAO method), in vivo	50, 100, and 150 mg/kg b.w. (p.o.)	↓ brain edema, neuroprotective, ↑ the integrity of the BBB, ↓ ischemic brain injury, ↓iNOS, ↓MCP-1, ↓COX-2 ↑Arg-1, ↑IL-10, ↑CD206	[128]
	Rats (focal cerebral ischemia: MCAO), in vivo	25, 37.5, and 50 mg/kg b.w	(-) unknown effects	[164]
	Human SH-SY5Y neuroblastoma cells, in vitro	IC_50_ = 0.1—1 μM	↓ ROS, ↓apoptosis ↓mitochondrial dysfunction ↓hypoxia,↑reoxygenation ↑protection from cerebral ischemia/reperfusion injury	
	Rats (permanent cerebral hypo-perfusion), in vivo	100 mg/kg b.w. (p.o.)	↑spatial memory performance ↑MDA ↓cognitive deficits through the elevation of cerebral antioxidant defense ↑activity against 2-vessel occlusion (2VO)	[87]
	Rats (permanent cerebral hypo-perfusion), in vivo	100 mg/kg b.w. (p.o.)	↑ flourished passive avoidance of memory, LTP in the HIP, and cell survival in the HIP and cortex of ischemic rats	[142]
	Rats (transient cerebral hypo-perfusion), in vivo	50, 100, and 200 mg/kg b.w. (p.o.)	↑antioxidant defense against BCCA occlusion ↑neuroprotection	[49]
Neuropathic pain	Mice (pain developed by paclitaxel: 2 mg/kg, i.p.), in vivo	20 and 40 mg/kg b.w	↓ TNF-α, ↓Ca^2+^, ↓TBARS, ↓superoxide anion, ↓GSH, ↓MPO, ↓ thermal and mechanical hyperalgesia	[82]
Brain tumor/Cerebral Glioblastoma	DBTRG-05MG human glioblastoma cells, in vitro	IC_50_ = 20—40 µM	↑Ca^2+^ ↑phospholipase C-dependent release from the ER ↑ROS, ↑apoptosis, ↑cytotoxicity	[68]
	T98G human glioblastoma cell lines, in vitro	IC_50_ = 100 µg/ml	↑miR-421 regulation of the cell cycle S-phase ↑serine/ threonine protein kinase ↑DNA damage ↑cell cycle arrest at the G1-S and S phases ↑apoptosis, ↑cytotoxicity	[119]
	U87, U251 human glioma cells, in vitro	IC_50_ = 20 μg/ml	↓glioma cells viability, ↓proliferation, ↓invasion, ↓angiogenesis, ↑cytotoxicity	[98]
Neuroinflammation	Sprague Dawley rats (neuroinflammation induced by intranigral infusion of LPS), in vivo	50 and 100 mg/kg b.w. (p.o.)	↓ iNOS, ↓IL-1β, ↓heme oxygenase-1 level, ↓α-synuclein aggregation, ↓caspase 3,↓ RIPK-1, ↓RIPK-3 levels, ↓ROS, ↓apoptosis	[95]
	LPS-treated BV2 microglial cells, in vitro	IC_50_ = 25– 100 μM	↓NO levels,↓ iNOS expression	
	Hippocampal neurons co-cultured with glial cells, (LPC-induced inflammation), in vitro	IC_50_ = 1.0 µM	↓NF-κB, ↓COX-2, ↓tenascin-C, ↓chondroitin sulfate proteoglycans and ↓glial fibrillary acidic protein	[156]
	Wistar rats (traumatic brain injury by Marmarou’s method), in vivo	100 mg/kg b.w. (p.o)	↓IL-1β, ↓IL-6, and ↓TNF-α	[142]
	BV-2 cells and Neuro-2A cells (treated by oligomeric Aβ), in vitro	IC_50_ = 5–50 µM	↓iNOS, ↓IL-1β, ↓COX-2, and ↓NF-kB	[85]

↑increase, ↓decrease, *GA* gallic acid, *AD* Alzheimer disease, *Aβ* amyloid β protein, *HIP* hippocampus, *ChEs* cholinesterase, *BACE-1* beta secretase-1, *i.p* intraperitoneally, *STZ* streptozotocin, *BDNF* brain-derived neurotrophic factor, *TMT* trimethyltin, *TNF- α* tumour necrosis factor-α, *SOD* superoxide dismutase, *GPx* glutathione peroxidase, *CAT* catalase, *TBARS*: 2-thiobarbituric acid reactive substances, κ-CN kappa-casein, *NF-kB* nuclear factor kappa B, *ICV* intracerebroventricular, *Nrf2* nuclear factor erythroid 2–related factor 2, *p-CREB* phosphorylated cAMP-responsive element binding protein, *6-OHDA*: 6-hydroxydopamine, *PD* Parkinson’s disease, *GSH* glutathione, *MDA* malondialdehyde, *TCA* tricarboxylic acid, *EPM* elevated plus maze, *5-HT*_*1A*_ 5-hydroxytryptamine (serotonin) receptor 1A, *TST* tail suspension test, *DST* despair swim test, *MAO-A* monoamine oxidase-A, *DP* dopamine, *AChE* acetyl cholinesterase, *GABA* gamma-aminobutyric acid, *MCAO* middle cerebral artery occlusion, *BBB* blood brain barrier, *MCP-1* monocyte chemoattractant protein-1, *COX-2* cyclooxygenase-2, *Arg-1* arginase-1, *IL-10* interleukin-10, *IL-6* interleukin-6, *IL-1β* interleukin-1β, *CD206* cluster of differentiation 206, *ROS* reactive oxygen species, *2VO*: 2-vessle occlusion, *BCCA* bilateral common carotid arteries, MPO myeloperoxidase, *ER* endoplasmic reticulum, *IC*_*50*_ half maximal inhibitory concentration, *RIPK-1* receptor-interacting protein kinase-1, *RIPK-3* receptor-interacting protein kinase-3, *LPS*: lipopolysaccharides, *NO* nitric oxide, *iNOS* inducible nitric oxide synthase, *LPC* lysolecithin, *LP* lipid peroxidation, *PPZ* perphenazine, HE hepatic encephalopathy, *BDL* bile duct ligation, *NOR* novel object recognition

## Clinical evidence

Clinical studies are an important step in discovering new treatments for diseases and the role of clinical trials is to inform about safety, accuracy, and evidence-based medicine [138, 147]. Recent years have seen a rise in clinical studies that make use of natural ingredients [28]. In recent years, a few clinical studies of GA-rich plants as a treatment for AD have been conducted [145]. NDs are tragically aided by oxidative stress, which causes free radicals to destroy brain cells. The toxicity of ROS plays a role in protein misfolding, glial cell activation, mitochondrial malfunction, and ultimately cellular demise [56]. So, the protective activity or antioxidant properties can prevent numerous NDs [106]. A human clinical study of GA for evaluating antioxidant value in diabetic patients (n = 19) found that GA diminished oxidized purines, pyrimidines, oxidized-LDL and C-reactive protein at 15 mg/person/day dose for 7 days of treatment resulting reduction of oxidative stress and this result suggests the capability of reflecting inflammation [51]. Another human intervention trial in Middle Europe by Ferk et al. [50] demonstrated that GA providing drinking water at a dose of 12.8 mg/p/d for 3 days to 16 volunteers reduced oxidized pyrimidines, purines, ROS and increased SOD, GPx as well as no alteration of total antioxidant capacity, MDA resulting reduction of oxidative damage [50].

## Toxicity and safety data

Toxicological studies are a crucial and necessary part of the drug development process, as they are carried out to assure that medications are safe for use in humans before they are tested on human subjects in clinical studies [125]. A variety of methods, including those using animals, in vitro studies utilizing cells/cell lines, and accidental chemical exposure, are used to determine toxicity [120, 148]. As a promising therapeutic candidate, the toxicity profile of GA has been the focus of many researchers. Preclinical experimental pharmacological studies in cell lines and in vivo models have shown that GA is devoid of toxicity and embryotoxicity even at low concentrations, but can be mildly toxic at high doses.

### In vitro toxicity

In the case of in vitro cytotoxicity, GA did not show any effect on the cell viability of neutrophils lower than 100 μM [62]. An in vitro study for evaluating the anti-Alzheimer activity of GA found that GA treatment on co-cultured Nerve-2A and BV-2 cells did not affect cell viability at 5–50 μM but higher concentrations (≥ 100 μM) are toxic [85].

### In vivo toxicity

An investigation for assessing acute toxicity of GA by Techer et al. [165] reported that GA was rationally non-toxic (96-h lethal concentration (LC_50_) > 100 mg/L) to the zebrafish within 100 mg/L dose [165]. In another in vivo experiment, oral administration of GA at a dose of 5 g/kg b.w. did not exhibit any signs and symptoms of toxicity or fatality in albino mice. GA, at 1000 mg/kg b.w., did not also significantly affect hematological markers in a subacute trial. This study summarises that if GA is taken orally, it is found to be non-toxic up to the dose of 5000 mg/kg b.w. [132]. A recent in vivo investigation demonstrated that the LD_50_ of GA in rabbits is 5000 mg/kg, however, the chronic toxicity of GA is uncertain from the study [44]. In a repetitive acute toxicity test of GA in albino mice for 28 days, it was reported that 900 mg/kg/d b.w. dose (p.o) did not generate any remarkable alteration in behavioral and morphological parameters of the mice and the result suggested that the LD_50_ of GA was found to be higher than 2000 mg/kg in mice [173]. In the case of GA derivatives, the acute oral toxicity of propyl gallate in rabbits, rats, hamsters, and mice has been shown to range from 2000 to 3800 mg/kg by the FAO/WHAO committee in 1962, 1965, 1974, and 1976 [118]. A study by Booth et al. [18] demonstrated that GA (80%) with some other herbal drugs (rhubarb, red sage, astragalus, turmeric and ginger) did not show any reproductive toxicity in pregnant rats at 430 mg/kg/d or below [18].

## Therapeutic perspectives and limitations

It has been found recently that the natural polyphenol antioxidant GA and its derivatives may have beneficial effects on health [99]. They provide protective activities against various NDs, inflammation and others due to their antioxidant capacity [86, 99], as they increase the antioxidant enzymes CAT and GPx [93]. GA provided strong evidence to become a lead compound against NDs based on different animal experiments. The most intriguing benefit has been reported to be an anti-Alzheimer drug by inhibiting Aβ formation [116]. Moreover, GA possesses the USFDA GRAS (generally recognized as safe) designation, indicating relatively low neurotoxicity and mortality at acute dosages in numerous animal models [84]. And literature demonstrated that its therapeutic window is large as it didn’t show toxicities up to 5000 mg/kg in different animal investigations [132]. Though the activities of GA are not widely clinically investigated, it provides a few positive potentials against various NDs and anticancer studies [78, 145].

Despite pharmacological evidence and scientific studies demonstrating the therapeutic benefits and safety of AG, its use is limited due to inadequate pharmacokinetic characteristics, including poor absorption, poor distribution, poor ability to cross the blood–brain barrier, low bioavailability, and rapid elimination. [48, 189]. Future measures are needed to enhance the bioavailability and biodegradability of this poorly water-soluble and non-biodegradable phenolic molecule. Also, no medicine contains gallic acid under testing or approval; as a result, future studies are needed regarding the development of nanoformulations and target release systems of AG. Another important therapeutic limitation is represented by the lack of translational pharmacological studies that accurately establish effective therapeutic doses in humans and the appropriate route of administration.

## Conclusion

This study focused on the in vitro and in vivo neurological effects of GA and its mechanism. It is apparent that GA and its derivatives function in a pivotal way to protect from various diseases, mainly because of their antioxidant feature, and this capability is essential to dealing with the neurodegeneration caused by oxidative stress and some other neuroprotection mechanisms. This review provides insights into the significant neurological effects of GA as a potential lead compound to treat various neurogenerative diseases, including brain tumors, glioblastoma therapy, cerebral ischemia/reperfusion, anxiety and memory loss, depression, sedation, psychosis, neuropathic pain, AD, and PD as well as neuroinflammation. The study also demonstrated that nanoformulations of GA may vastly increase the bioavailability and efficacy through the crossing of the BBB, which may be considered a new technique to treat neurological diseases with GA. In future, more extensive clinical studies on the neurobiological effects of GA are required to establish its efficacy for long-term use in the field of neurological diseases.

## Data Availability

Not applicable.

## References

[1] (PAHO), P. A. H. O. 2022. *Burden of Neurological Conditions*. PAHO. https://www.paho.org/en/enlace/burden-neurological-conditions. Accessed Sep 30 2022.

[2] Abílio VC, Araujo CC, Bergamo M, Calvente PR, D'Almeida V, Ribeiro RDA, Frussa-Filho R (2003). Vitamin E attenuates reserpine-induced oral dyskinesia and striatal oxidized glutathione/reduced glutathione ratio (GSSG/GSH) enhancement in rats. Prog Neuro Psychopharmacol Biol Psychiatry.

[3] Akhtar A, Kamal AK (2013). Evidence based medicine review: benefit of neuroprotection in acute ischaemic stroke, shall we dare to hope? *JPMA*. J Pak Med Assoc.

[4] Alexander BM, Cloughesy TF (2017). Adult glioblastoma. J Clin Oncol.

[5] Aloizou AM, Siokas V, Pateraki G, Liampas I, Bakirtzis C, Tsouris Z, Lazopoulos G, Calina D, Docea AO, Tsatsakis A, Bogdanos DP, Dardiotis E (2021). Thinking outside the ischemia box: advancements in the use of multiple sclerosis drugs in ischemic stroke. J Clin Med.

[6] Andreassen OA, Jørgensen HA (2000). Neurotoxicity associated with neuroleptic-induced oral dyskinesias in rats: implications for tardive dyskinesia?. Prog Neurobiol.

[7] Asangani IA, Rasheed SA, Nikolova D, Leupold J, Colburn N, Post S, Allgayer H (2008). MicroRNA-21 (miR-21) post-transcriptionally downregulates tumor suppressor Pdcd4 and stimulates invasion, intravasation and metastasis in colorectal cancer. Oncogene.

[8] Asgharian P, Quispe C, Herrera-Bravo J, Sabernavaei M, Hosseini K, Forouhandeh H, Ebrahimi T, Sharafi-Badr P, Tarhriz V, Soofiyani SR, Helon P, Rajkovic J, Durna Daştan S, Docea AO, Sharifi-Rad J, Calina D, Koch W, Cho WC (2022). Pharmacological effects and therapeutic potential of natural compounds in neuropsychiatric disorders: an update. Front Pharmacol.

[9] Atri A (2019). Current and future treatments in Alzheimer’s disease. Semin Neurol.

[10] Backonja M-M (2002). Use of anticonvulsants for treatment of neuropathic pain. Neurology.

[11] Badhani B, Sharma N, Kakkar R (2015). Gallic acid: a versatile antioxidant with promising therapeutic and industrial applications. RSC Adv.

[12] Bai J, Zhang Y, Tang C, Hou Y, Ai X, Chen X, Zhang Y, Wang X, Meng X (2021). Gallic acid: Pharmacological activities and molecular mechanisms involved in inflammation-related diseases. Biomed Pharmacother.

[13] Barcelos RCS, Benvegnú DM, Boufleur N, Pase C, Teixeira AM, Reckziegel P, Emanuelli T, Da Rocha JBT, Bürger ME (2011). Short term dietary fish oil supplementation improves motor deficiencies related to reserpine-induced parkinsonism in rats. Lipids.

[14] Bayramoglu G, Kurt H, Bayramoglu A, Gunes HV, Degirmenci İ, Colak S (2015). Preventive role of gallic acid on hepatic ischemia and reperfusion injury in rats. Cytotechnology.

[15] Baziyar Y, Edalatmanesh MA, Hosseini SA, Zar A (2016). The effects of endurance training and gallic acid on BDNF and TNF-a in male rats with Alzheimer. Int J Appl Exerc Physiol.

[16] Bhattacharyya S, Ahammed SM, Saha BP, Mukherjee PK (2013). The gallic acid–phospholipid complex improved the antioxidant potential of gallic acid by enhancing its bioavailability. AAPS Pharm Sci Tech.

[17] Bjerkvig R, Lund-Johansen M, Edvardsen K (1997). Tumor cell invasion and angiogenesis in the central nervous system. Curr Opin Oncol.

[18] Booth A, Amen RJ, Scott M, Greenway FL (2010). Oral dose-ranging developmental toxicity study of an herbal supplement (NT) and gallic acid in rats. Adv Ther.

[19] Brown TB, Lovato LM, Parker D (2005). Procedural sedation in the acute care setting. Am Fam Phys.

[20] Bubeníková-Valešová V, Horáček J, Vrajová M, Höschl C (2008). Models of schizophrenia in humans and animals based on inhibition of NMDA receptors. Neurosci Biobehav Rev.

[21] Busanello A, Barbosa NBV, Peroza LR, Farias LE, Burger ME, Barreto KP, Fachinetto R (2011). Resveratrol protects against a model of vacuous chewing movements induced by reserpine in mice. Behav Pharmacol.

[22] Calina D, Buga AM, Mitroi M, Buha A, Caruntu C, Scheau C, Bouyahya A, El Omari N, El Menyiy N, Docea AO (2020). The treatment of cognitive, behavioural and motor impairments from brain injury and neurodegenerative diseases through cannabinoid system modulation-evidence from in vivo studies. J Clin Med.

[23] Calina D, Buga AM, Mitroi M, Buha A, Caruntu C, Scheau C, Bouyahya A, El Omari N, El Menyiy N, Docea AO (2020). The treatment of cognitive, behavioural and motor impairments from brain injury and neurodegenerative diseases through cannabinoid system modulation—evidence from in vivo studies. J Clin Med.

[24] Calina D, Docea AO, Golokhvast KS, Sifakis S, Tsatsakis A, Makrigiannakis A (2019). Management of endocrinopathies in pregnancy: a review of current evidence. Int J Environ Res Public Health.

[25] Can ÖD, Turan N, Özkay ÜD, Öztürk Y (2017). Antidepressant-like effect of gallic acid in mice: dual involvement of serotonergic and catecholaminergic systems. Life Sci.

[26] Castro JP, Frussa-Filho R, Fukushiro DF, Silva RH, Medrano WA, Ribeiro RDA, Abílio VC (2006). Effects of baclofen on reserpine-induced vacuous chewing movements in mice. Brain Res Bull.

[27] Chandrasekhar Y, Phani Kumar G, Ramya E, Anilakumar K (2018). Gallic acid protects 6-OHDA induced neurotoxicity by attenuating oxidative stress in human dopaminergic cell line. Neurochem Res.

[28] Chaudhary P, Mitra D, Das Mohapatra PK, Oana Docea A, Mon Myo E, Janmeda P, Martorell M, Iriti M, Ibrayeva M, Sharifi-Rad J, Santini A, Romano R, Calina D, Cho WC (2023). Camellia sinensis: insights on its molecular mechanisms of action towards nutraceutical, anticancer potential and other therapeutic applications. Arab J Chem.

[29] Chen H, Yoshioka H, Kim GS, Jung JE, Okami N, Sakata H, Maier CM, Narasimhan P, Goeders CE, Chan PH (2011). Oxidative stress in ischemic brain damage: mechanisms of cell death and potential molecular targets for neuroprotection. Antioxid Redox Signal.

[30] Chhillar R, Dhingra D (2013). Antidepressant-like activity of gallic acid in mice subjected to unpredictable chronic mild stress. Fundam Clin Pharmacol.

[31] Chorpita BF, Barlow DH (1998). The development of anxiety: the role of control in the early environment. Psychol Bull.

[32] Choubey S, Varughese LR, Kumar V, Beniwal V (2015). Medicinal importance of gallic acid and its ester derivatives: a patent review. Pharma Patent Analyst.

[33] Chowdhury MR, Moshikur RM, Wakabayashi R, Tahara Y, Kamiya N, Moniruzzaman M, Goto M (2018). Ionic-liquid-based paclitaxel preparation: a new potential formulation for cancer treatment. Mol Pharm.

[34] Christophe M, Nicolas S (2006). Mitochondria: a target for neuroprotective interventions in cerebral ischemia-reperfusion. Curr Pharm Des.

[35] Cioboată R, Găman A, Traşcă D, Ungureanu A, Docea AO, Tomescu P, Gherghina F, Arsene AL, Badiu C, Tsatsakis AM, Spandidos DA, Drakoulis N, Călina D (2017). Pharmacological management of non-alcoholic fatty liver disease: atorvastatin versus pentoxifylline. Exp Ther Med.

[36] Coppen A (1967). The biochemistry of affective disorders. Br J Psychiatry.

[37] Custódio L, Vizetto-Duarte C, Cebeci F, Özçelik B, Sharopov F, Gürer ES, Kumar M, Iriti M, Sharifi-Rad J, Calina D (2023). Natural products of relevance in the management of attention deficit hyperactivity disorder. eFood.

[38] Daglia M, Di Lorenzo A, Nabavi SF, Talas ZS, Nabavi MS (2014). Polyphenols: well beyond the antioxidant capacity: gallic acid and related compounds as neuroprotective agents: you are what you eat!. Curr Pharma Biotechnol.

[39] Dauer W, Przedborski S (2003). Parkinson's disease: mechanisms and models. Neuron.

[40] Davie CA (2008). A review of Parkinson’s disease. Br Med Bull.

[41] Davis ME (2016). Glioblastoma: overview of disease and treatment. Clin J Oncol Nurs.

[42] Dhingra D, Chhillar R, Gupta A (2012). Antianxiety-like activity of gallic acid in unstressed and stressed mice: possible involvement of nitriergic system. Neurochem Res.

[43] Dickson DW (2012). Parkinson’s disease and parkinsonism: neuropathology. Cold Spring Harb Perspect Med.

[44] Dollahite JW, Pigeon RF, Camp BJ (1962). The toxicity of gallic acid, pyrogallol, tannic acid, and Quercus havardi in the rabbit. Am J Vet Res.

[45] Doyle T, Chen Z, Muscoli C, Bryant L, Esposito E, Cuzzocrea S, Dagostino C, Ryerse J, Rausaria S, Kamadulski A (2012). Targeting the overproduction of peroxynitrite for the prevention and reversal of paclitaxel-induced neuropathic pain. J Neurosci.

[46] Dwivedi N, Shah J, Mishra V, Tambuwala M, Kesharwani P (2019). Nanoneuromedicine for management of neurodegenerative disorder. J Drug Deliv Sci Technol.

[47] Faludi G, Dome P, Lazary J (2011). Origins and perspectives of schizophrenia research. Neuropsychopharmacol Hung.

[48] Faralli A, Shekarforoush E, Mendes AC, Chronakis IS (2019). Enhanced transepithelial permeation of gallic acid and (−)-epigallocatechin gallate across human intestinal caco-2 cells using electrospun xanthan nanofibers. Pharmaceutics.

[49] Farbood Y, Sarkaki A, Hashemi S, Mansouri MT, Dianat M (2013). The effects of gallic acid on pain and memory following transient global ischemia/reperfusion in Wistar rats. Avic J Phytomed.

[50] Ferk F, Chakraborty A, Jäger W, Kundi M, Bichler J, Mišík M, Wagner K-H, Grasl-Kraupp B, Sagmeister S, Haidinger G (2011). Potent protection of gallic acid against DNA oxidation: results of human and animal experiments. Mutat Res.

[51] Ferk F, Kundi M, Brath H, Szekeres T, Al-Serori H, Mišík M, Saiko P, Marculescu R, Wagner KH, Knasmueller S (2018). Gallic acid improves health-associated biochemical parameters and prevents oxidative damage of DNA in type 2 diabetes patients: Results of a placebo-controlled pilot study. Mol Nutr Food Res.

[52] Fernandes FHA, Salgado HRN (2016). Gallic acid: review of the methods of determination and quantification. Crit Rev Anal Chem.

[53] Ferrazzano GF, Amato I, Ingenito A, Zarrelli A, Pinto G, Pollio A (2011). Plant polyphenols and their anti-cariogenic properties: a review. Molecules.

[54] Fibiger HC (1991). Cholinergic mechanisms in learning, memory and dementia: a review of recent evidence. Trends Neurosci.

[55] Fu Y, Yang J, Wang X, Yang P, Zhao Y, Li K, Chen Y, Xu Y (2018). Herbal compounds play a role in neuroprotection through the inhibition of microglial activation. J Immunol Res.

[56] Fulda S, Gorman AM, Hori O, Samali A (2010). Cellular stress responses: cell survival and cell death. Int J Cell Biol.

[57] Gribkoff VK, Kaczmarek LK (2017). The need for new approaches in CNS drug discovery: why drugs have failed, and what can be done to improve outcomes. Neuropharmacology.

[58] Griffiths LA, Duggett NA, Pitcher AL, Flatters SJ (2018). Evoked and ongoing pain-like behaviours in a rat model of paclitaxel-induced peripheral neuropathy. Pain Res Manage.

[59] Grotta JC, Albers GW, Broderick JP, Kasner SE, Lo EH, Sacco RL, Wong LK, Day AL (2021). Stroke e-book: pathophysiology, diagnosis, and management.

[60] Hajipour S, Sarkaki A, Farbood Y, Eidi A, Mortazavi P, Valizadeh Z (2016). Effect of gallic acid on dementia type of Alzheimer disease in rats: electrophysiological and histological studies. Basic Clin Neurosci.

[61] Haslam E, Cai Y (1994). Plant polyphenols (vegetable tannins): gallic acid metabolism. Nat Prod Rep.

[62] Haute GV, Caberlon E, Squizani E, De Mesquita FC, Pedrazza L, Martha BA, Da Silva Melo DA, Cassel E, Czepielewski RS, Bitencourt S (2015). Gallic acid reduces the effect of LPS on apoptosis and inhibits the formation of neutrophil extracellular traps. Toxicol Vitro.

[63] Hemphill Iii JC, Greenberg SM, Anderson CS, Becker K, Bendok BR, Cushman M, Fung GL, Goldstein JN, Macdonald RL, Mitchell PH (2015). Guidelines for the management of spontaneous intracerebral hemorrhage: a guideline for healthcare professionals from the American heart association/American stroke association. Stroke.

[64] Hiyoshi Y, Kamohara H, Karashima R, Sato N, Imamura Y, Nagai Y, Yoshida N, Toyama E, Hayashi N, Watanabe M (2009). Microrna-21 regulates the proliferation and invasion in esophageal squamous cell carcinoma. Clin Cancer Res.

[65] Hossain MA, Weli AM, Ahmed SHI (2019). Comparison of total phenols, flavonoids and antioxidant activity of various crude extracts of hyoscyamus gallagheri traditionally used for the treatment of epilepsy. Clin Phytosci.

[66] Hossmann K-A (2006). Pathophysiology and therapy of experimental stroke. Cell Mol Neurobiol.

[67] Houshmand G, Nikbakht J, Mahmoudi M, Assadpour S, Arab FA, Arimi A, Almasian E (2022). Evaluation of gallic acid effect on perphenazine induced catatonia in rats evaluating the effect of gallic acid on perphenazine induced catatonia in rats. Armaghane Danesh.

[68] Hsu S-S, Chou C-T, Liao W-C, Shieh P, Kuo D-H, Kuo C-C, Jan C-R, Liang W-Z (2016). The effect of gallic acid on cytotoxicity, Ca2+ homeostasis and ROS production in DBTRG-05MG human glioblastoma cells and CTX TNA2 rat astrocytes. Chem Biol Interact.

[69] Hwang D-S, Kim HG, Kwon H-J, Cho J-H, Lee C-H, Lee J-M, Jang J-B, Kim Y-S, Lee K-S, Oh MS (2011). Dangguijakyak-san, a medicinal herbal formula, protects dopaminergic neurons from 6-hydroxydopamine-induced neurotoxicity. J Ethnopharmacol.

[70] Islam MS, Quispe C, Hossain R, Islam MT, Al-Harrasi A, Al-Rawahi A, Martorell M, Mamurova A, Seilkhan A, Altybaeva N, Abdullayeva B, Docea AO, Calina D, Sharifi-Rad J (2021). Neuropharmacological effects of quercetin: a literature-based review. Front Pharmacol.

[71] Jafaripour L, Esmaeilpour K, Maneshian M, Bashiri H, Rajizadeh MA, Ahmadvand H, Asadi-Shekaari M (2021). The effect of gallic acid on memory and anxiety-like behaviors in rats with bile duct ligation-induced hepatic encephalopathy: role of AMPK pathway. Avic J Phytomed.

[72] Janoutová J, Serý O, Hosák L, Janout V (2015). Is mild cognitive impairment a precursor of Alzheimer's disease? short review. Cent Eur J Public Health.

[73] Javad Sharifi-Rad ZMA, Adetunji CO, Michael OS, Chandran D, Radha R, Sharma N, Kumar M, Calina D (2022). Neuroprotective effect of curcumin and curcumin-integrated nanocarriers in stroke: from mechanisms to therapeutic opportunities. Minerva Biotechnol and Biomol Res.

[74] Jee Y-S, Ko I-G, Sung Y-H, Lee J-W, Kim Y-S, Kim S-E, Kim B-K, Seo J-H, Shin M-S, Lee H-H (2008). Effects of treadmill exercise on memory and c-Fos expression in the hippocampus of the rats with intracerebroventricular injection of streptozotocin. Neurosci Lett.

[75] Jha MK, Jeon S, Suk K (2012). Glia as a link between neuroinflammation and neuropathic pain. Immune Net.

[76] Jiang X-W, Bai J-P, Zhang Q, Hu X-L, Tian X, Zhu J, Liu J, Meng W-H, Zhao Q-C (2017). Caffeoylquinic acid derivatives protect SH-SY5Y neuroblastoma cells from hydrogen peroxide-induced injury through modulating oxidative status. Cell Mol Neurobiol.

[77] Jiang X, Ganesan P, Rengarajan T, Choi D-K, Arulselvan P (2018). Cellular phenotypes as inflammatory mediators in Parkinson’s disease: interventional targets and role of natural products. Biomed Pharmacother.

[78] Jiang Y, Pei J, Zheng Y, Miao Y-J, Duan B-Z, Huang L-F (2021). Gallic acid: a potential anti-cancer agent. Chin J Integrat Med.

[79] Kang N, Lee J-H, Lee W, Ko J-Y, Kim E-A, Kim J-S, Heu M-S, Kim GH, Jeon Y-J (2015). Gallic acid isolated from Spirogyra sp. improves cardiovascular disease through a vasorelaxant and antihypertensive effect. Environ Toxicol Pharmacol.

[80] Karve IP, Taylor JM, Crack PJ (2016). The contribution of astrocytes and microglia to traumatic brain injury. Br J Pharmacol.

[81] Kasture VS, Katti SA, Mahajan D, Wagh R, Mohan M, Kasture SB (2009). Antioxidant and antiparkinson activity of gallic acid derivatives. Pharmacologyonline.

[82] Kaur S, Muthuraman A (2019). Ameliorative effect of gallic acid in paclitaxel-induced neuropathic pain in mice. Toxicol Rep.

[83] Kaye AD, Cornett EM, Hart B, Patil S, Pham A, Spalitta M, Mancuso KF (2018). Novel pharmacological nonopioid therapies in chronic pain. Curr Pain Headache Rep.

[84] Khan BA, Mahmood T, Menaa F, Shahzad Y, Yousaf AM, Hussain T, Ray SD (2018). New perspectives on the efficacy of gallic acid in cosmetics & nanocosmeceuticals. Curr Pharm Des.

[85] Kim MJ, Seong AR, Yoo JY, Jin CH, Lee YH, Kim YJ, Lee J, Jun WJ, Yoon HG (2011). Gallic acid, a histone acetyltransferase inhibitor, suppresses β-amyloid neurotoxicity by inhibiting microglial-mediated neuroinflammation. Mol Nutr Food Res.

[86] Kim Y-J (2007). Antimelanogenic and antioxidant properties of gallic acid. Biol Pharm Bull.

[87] Korani MS, Farbood Y, Sarkaki A, Moghaddam HF, Mansouri MT (2014). Protective effects of gallic acid against chronic cerebral hypoperfusion-induced cognitive deficit and brain oxidative damage in rats. Eur J Pharmacol.

[88] Kuipers SD, Bramham CR (2006). Brain-derived neurotrophic factor mechanisms and function in adult synaptic plasticity: new insights and implications for therapy. Curr Opin Drug Discov Devel.

[89] Kumar A, Yadav M, Parle M, Dhingra S, Dhull DK (2017). Potential drug targets and treatment of schizophrenia. Inflammopharmacology.

[90] Kumar N, Goel N (2019). Phenolic acids: Natural versatile molecules with promising therapeutic applications. Biotechnol Rep.

[91] Kuramatsu JB, Gerner ST, Huttner HB, Schwab S (2017). Acute management of anticoagulation-associated intracerebral hemorrhage. Neurol Int Open.

[92] Lal D, Gardner JJ (2012). Production, characterization and purification of tannase from Aspergillus niger. Eur J Exp Biol.

[93] Li L, Ng T, Gao W, Li W, Fu M, Niu S, Zhao L, Chen R, LIU, F.  (2005). Antioxidant activity of gallic acid from rose flowers in senescence accelerated mice. Life Sci.

[94] Lin AM, Fang S, Chao P, Yang C (2007). Melatonin attenuates arsenite-induced apoptosis in rat brain: involvement of mitochondrial and endoplasmic reticulum pathways and aggregation of α-synuclein. J Pineal Res.

[95] Liu Y-L, Hsu C-C, Huang H-J, Chang C-J, Sun S-H, Lin AM-Y (2020). Gallic acid attenuated LPS-induced neuroinflammation: protein aggregation and necroptosis. Mol Neurobiol.

[96] Liu Y, Pukala TL, Musgrave IF, Williams DM, Dehle FC, Carver JA (2013). Gallic acid is the major component of grape seed extract that inhibits amyloid fibril formation. Bioorg Med Chem Lett.

[97] Lotharius J, Brundin P (2002). Pathogenesis of Parkinson's disease: dopamine, vesicles and α-synuclein. Nat Rev Neurosci.

[98] Lu Y, Jiang F, Jiang H, Wu K, Zheng X, Cai Y, Katakowski M, Chopp M, To S-ST (2010). Gallic acid suppresses cell viability, proliferation, invasion and angiogenesis in human glioma cells. Eur J Pharm.

[99] Lu Z, Nie G, Belton PS, Tang H, Zhao B (2006). Structure–activity relationship analysis of antioxidant ability and neuroprotective effect of gallic acid derivatives. Neurochem Int.

[100] Mah L, Binns MA, Steffens DC, Initiative ASDN (2015). Anxiety symptoms in amnestic mild cognitive impairment are associated with medial temporal atrophy and predict conversion to Alzheimer disease. Am J Geriatr Psychiatry.

[101] Manoharan S, Guillemin GJ, Abiramasundari RS, Essa MM, Akbar M, Akbar MD (2016). The role of reactive oxygen species in the pathogenesis of Alzheimer’s disease, Parkinson’s disease, and Huntington’s disease: a mini review. Oxid Med Cell Longev.

[102] Manosroi A, Jantrawut P, Akazawa H, Akihisa T, Manosroi W, Manosroi J (2011). Transdermal absorption enhancement of gel containing elastic niosomes loaded with gallic acid from terminalia chebula galls. Pharm Biol.

[103] Mansouri MT, Farbood Y, Sameri MJ, Sarkaki A, Naghizadeh B, Rafeirad M (2013). Neuroprotective effects of oral gallic acid against oxidative stress induced by 6-hydroxydopamine in rats. Food Chem.

[104] Mansouri MT, Naghizadeh B, Ghorbanzadeh B, Farbood Y, Sarkaki A, Bavarsad K (2013). Gallic acid prevents memory deficits and oxidative stress induced by intracerebroventricular injection of streptozotocin in rats. Pharmacol Biochem Behav.

[105] Mansouri MT, Soltani M, Naghizadeh B, Farbood Y, Mashak A, Sarkaki A (2014). A possible mechanism for the anxiolytic-like effect of gallic acid in the rat elevated plus maze. Pharmacol Biochem Behav.

[106] Mititelu RR, Padureanu R, Bacanoiu M, Padureanu V, Docea AO, Calina D, Barbulescu AL, Buga AM (2020). Inflammatory and oxidative stress markers-mirror tools in rheumatoid arthritis. Biomedicines.

[107] Monji A, Kato T, Kanba S (2009). Cytokines and schizophrenia: Microglia hypothesis of schizophrenia. Psychiatry Clin Neurosci.

[108] Nabavi SF, Habtemariam S, Di Lorenzo A, Sureda A, Khanjani S, Nabavi SM, Daglia M (2016). Post-stroke depression modulation and in vivo antioxidant activity of gallic acid and its synthetic derivatives in a murine model system. Nutrients.

[109] Nagai T, Kitahara Y, Shiraki A, Hikita T, Taya S, Kaibuchi K, Yamada K (2010). Dysfunction of dopamine release in the prefrontal cortex of dysbindin deficient sandy mice: an in vivo microdialysis study. Neurosci Lett.

[110] Nagpal K, Singh S, Mishra D (2013). Optimization of brain targeted gallic acid nanoparticles for improved antianxiety-like activity. Int J Biol Macromol.

[111] Nagpal K, Singh SK, Mishra DN (2012). Nanoparticle mediated brain targeted delivery of gallic acid: in vivo behavioral and biochemical studies for improved antioxidant and antidepressant-like activity. Drug Deliv.

[112] Navya K, Kumar GP, Anilakumar K (2017). Ameliorating effect of curculigo orchoides on chromium (VI) induced oxidative stress via, modulation of cytokines, transcription factors and apoptotic genes. J Appl Biomed.

[113] Nayeem N, Asdaq S, Salem H, Ahel-Alfqy S (2016). Gallic acid: a promising lead molecule for drug development. J Appl Pharm.

[114] Nelson L, Guo T, Lu J, Saper C, Franks N, Maze M (2002). The sedative component of anesthesia is mediated by GABAA receptors in an endogenous sleep pathway. Nat Neurosci.

[115] Ogunlade B, Adelakun S, Agie J (2022). Nutritional supplementation of gallic acid ameliorates Alzheimer-type hippocampal neurodegeneration and cognitive impairment induced by aluminum chloride exposure in adult Wistar rats. Drug Chem Toxicol.

[116] Ogunsuyi OB, Oboh G, Oluokun OO, Ademiluyi AO, Ogunruku OO (2020). Gallic acid protects against neurochemical alterations in transgenic drosophila model of Alzheimer’s disease. Adv Tradit Med.

[117] Ooyama K, Wu J, Nosaka N, Aoyama T, Kasai M (2008). Combined intervention of medium-chain triacylglycerol diet and exercise reduces body fat mass and enhances energy expenditure in rats. J Nutr Sci Vitaminol.

[118] Pal SM, Avneet G, Siddhraj SS (2018). Gallic acid: pharmacogical promising lead molecule: a review. Int J Pharmacogn Phytochem Res.

[119] Paolini A, Curti V, Pasi F, Mazzini G, Nano R, Capelli E (2015). Gallic acid exerts a protective or an anti-proliferative effect on glioma T98G cells via dose-dependent epigenetic regulation mediated by miRNAs. Int J Oncol.

[120] Parasuraman S (2011). Toxicological screening. J Pharmacol Pharmacother.

[121] Park HJ (2014). Chemotherapy induced peripheral neuropathic pain. Korean J Anesthesiol.

[122] Parle M, Sharma K (2013). Biomarker and causative factor of schizophrenia. Int Res J Pharm.

[123] Patil P, Killedar S (2021). Improving gallic acid and quercetin bioavailability by polymeric nanoparticle formulation. Drug Dev Ind Pharm.

[124] Pereira MM, De Morais H, Dos Santos Silva E, Corso CR, Adami ER, Carlos RM, Acco A, Zanoveli JM (2018). The antioxidant gallic acid induces anxiolytic-, but not antidepressant-like effect, in streptozotocin-induced diabetes. Metab Brain Dis.

[125] Prasher P, Sharma M, Sharma AK, Sharifi-Rad J, Calina D, Hano C, Cho WC (2023). Key oncologic pathways inhibited by erinacine a: a perspective for its development as an anticancer molecule. Biomed Pharmacother.

[126] Pubchem. 2022a. Gallic acid. PubChem. https://pubchem.ncbi.nlm.nih.gov/compound/Gallic-acid. Accessed September 30 2022.

[127] Pubchem. 2022b. PubChem. https://pubchem.ncbi.nlm.nih.gov/. Accessed.

[128] Qu Y, Wang L, Mao Y (2022). Gallic acid attenuates cerebral ischemia/re-perfusion-induced blood–brain barrier injury by modifying polarization of microglia. J Immunotoxicol.

[129] Quispe C, Herrera-Bravo J, Javed Z, Khan K, Raza S, Gulsunoglu-Konuskan Z, Daştan SD, Sytar O, Martorell M, Sharifi-Rad J, Calina D (2022). Therapeutic applications of curcumin in diabetes: a review and perspective. Biomed Res Int.

[130] Quispe C, Herrera-Bravo J, Khan K, Javed Z, Semwal P, Painuli S, Kamiloglu S, Martorell M, Calina D, Sharifi-Rad J (2022). Therapeutic applications of curcumin nanomedicine formulations in cystic fibrosis. Prog Biomat.

[131] Rahaman MM, Hossain R, Herrera-Bravo J, Islam MT, Atolani O, Adeyemi OS, Owolodun OA, Kambizi L, Daştan SD, Calina D, Sharifi-Rad J (2023). Natural antioxidants from some fruits seeds foods natural products and associated health benefits: an update. Food Sci Nutrition.

[132] Rajalakshmi K, Devaraj H, Devaraj SN (2001). Assessment of the no-observed-adverse-effect level (NOAEL) of gallic acid in mice. Food Chem Toxicol.

[133] Ratheesh G, Tian L, Venugopal JR, Ezhilarasu H, Sadiq A, Fan TP, Ramakrishna S (2017). Role of medicinal plants in neurodegenerative diseases. Biomanufact Rev.

[134] Reckziegel P, Peroza LR, Schaffer LF, Ferrari MC, De Freitas CM, Bürger ME, Fachinetto R (2013). Gallic acid decreases vacuous chewing movements induced by reserpine in rats. Pharmacol Biochem Behavior.

[135] Reichel A, Lienau P (2015). Pharmacokinetics in drug discovery: an exposure-centred approach to optimising and predicting drug efficacy and safety. New Appr Drug Discovery.

[136] Ressler KJ, Nemeroff CB (2000). Role of serotonergic and noradrenergic systems in the pathophysiology of depression and anxiety disorders. Depress Anxiety.

[137] Salehi A, Rabiei Z, Setorki M (2018). Effect of gallic acid on chronic restraint stress-induced anxiety and memory loss in male BALB/c mice. Iran J Basic Med Sci.

[138] Salehi B, Prakash Mishra A, Nigam M, Karazhan N, Shukla I, Kiełtyka-Dadasiewicz A, Sawicka B, Głowacka A, Abu-Darwish MS, Hussein Tarawneh A, Gadetskaya AV (2021). Ficus plants: State of the art from a phytochemical, pharmacological, and toxicological perspective. Phytother Res.

[139] Salehi B, Sestito S, Rapposelli S, Peron G, Calina D, Sharifi-Rad M, Sharopov F, Martins N, Sharifi-Rad J (2019). Epibatidine: a promising natural alkaloid in health. Biomolecules.

[140] Samad N, Jabeen S, Imran I, Zulfiqar I, Bilal K (2019). Protective effect of gallic acid against arsenic-induced anxiety−/depression-like behaviors and memory impairment in male rats. Metab Brain Dis.

[141] Sameri MJ, Sarkaki A, Farbood Y, Mansouri S (2011). Motor disorders and impaired electrical power of pallidal EEG improved by gallic acid in animal model of Parkinson's disease. Pakistan Journal of Biological Sciences: PJBS.

[142] Sarkaki A, Farbood Y, Gharib-Naseri MK, Badavi M, Mansouri MT, Haghparast A, Mirshekar MA (2015). Gallic acid improved behavior, brain electrophysiology, and inflammation in a rat model of traumatic brain injury. Can J Physiol Pharmacol.

[143] Schildkraut JJ (1965). The catecholamine hypothesis of affective disorders: a review of supporting evidence. Am J Psychiatry.

[144] Selkoe DJ (1991). The molecular pathology of Alzheimer's disease. Neuron.

[145] Shabani S, Rabiei Z, Amini-Khoei H (2020). Exploring the multifaceted neuroprotective actions of gallic acid: A review. Int J Food Prop.

[146] Shahrzad S, Aoyagi K, Winter A, Koyama A, Bitsch I (2001). Pharmacokinetics of gallic acid and its relative bioavailability from tea in healthy humans. J Nutr.

[147] Sharifi-Rad J, Dey A, Koirala N, Shaheen S, El Omari N, Salehi B, Goloshvili T, Cirone Silva NC, Bouyahya A, Vitalini S, Varoni EM (2021). Cinnamomum species: bridging phytochemistry knowledge, pharmacological properties and toxicological safety for health benefits. Front Pharmacol.

[148] Sharifi-Rad J, Kamiloglu S, Yeskaliyeva B, Beyatli A, Alfred MA, Salehi B, Calina D, Docea AO, Imran M, Kumar NVA, Romero-Roman ME, Maroyi A, Martorell M (2020). Pharmacological activities of psoralidin: a comprehensive review of the molecular mechanisms of action. Front Pharmacol.

[149] Sharifi-Rad J, Quispe C, Herrera-Bravo J, Martorell M, Sharopov F, Tumer TB, Kurt B, Lankatillake C, Docea AO, Moreira AC, Dias DA, Mahomoodally MF, Lobine D, Cruz-Martins N, Kumar M, Calina D (2021). A Pharmacological perspective on plant-derived bioactive molecules for epilepsy. Neurochem Res.

[150] Sharifi-Rad J, Quispe C, Patra JK, Singh YD, Panda MK, Das G, Adetunji CO, Michael OS, Sytar O, Polito L, Živković J (2021). Paclitaxel: application in modern oncology and nanomedicine-based cancer therapy. Oxidative Med Cell Long.

[151] Sharifi-Rad J, Rapposelli S, Sestito S, Herrera-Bravo J, Arancibia-Diaz A, Salazar LA, Yeskaliyeva B, Beyatli A, Leyva-Gómez G, González-Contreras C, Gürer ES, Martorell M, Calina D (2022). Multi-target mechanisms of phytochemicals in Alzheimer’s disease: effects on oxidative stress neuroinflammation and protein aggregation. J Pers Med.

[152] Sharifi-Rad J, Sharopov F, Ezzat SM, Zam W, Ademiluyi AO, Oyeniran OH, Adetunji CO, Roli OI, Živković J, Martorell M, Docea AO, El Omari N, Bouyahya A, Lorenzo JM, Calina D (2023). An updated review on glycoprotein IIb/IIIa inhibitors as antiplatelet agents: basic and clinical perspectives. High Blood Press Cardiovasc Prev.

[153] Sharma E, Attri DC, Sati P, Dhyani P, Szopa A, Sharifi-Rad J, Hano C, Calina D, Cho WC (2022). Recent updates on anticancer mechanisms of polyphenols. Front Cell Devel Biol.

[154] Shi L, Cheng Z, Zhang J, Li R, Zhao P, Zhen F, You Y (2008). hsa-mir-181a and hsa-mir-181b function as tumor suppressors in human glioma cells. Brain Res.

[155] Shukla S, Singh B, Singh A, Singh C (2022). Emerging and advanced drug delivery systems for improved biopharmaceutical attributes of gallic acid: a review. Phytomed Plus.

[156] Siddiqui S, Kamal A, Khan F, Jamali KS, Saify ZS (2019). Gallic and vanillic acid suppress inflammation and promote myelination in an in vitro mouse model of neurodegeneration. Mol Biol Rep.

[157] Silbergeld DL, Chicoine MR (1997). Isolation and characterization of human malignant glioma cells from histologically normal brain. J Neurosurg.

[158] Smith L, Anwar A, Fragen M, Rananto C, Johnson R, Holbert D (2000). Cytokines and cell adhesion molecules associated with high-intensity eccentric exercise. Eur J Appl Physiol.

[159] Snyder SH, Banerjee SP, Yamamura HI, Greenberg D (1974). Drugs, Neurotransmitters, and Schizophrenia: Phenothiazines, amphetamines, and enzymes synthesizing psychotomimetic drugs aid schizophrenia research. Science.

[160] Solomon A, Mangialasche F, Richard E, Andrieu S, Bennett DA, Breteler M, Fratiglioni L, Hooshmand B, Khachaturian AS, Schneider LS (2014). Advances in the prevention of Alzheimer's disease and dementia. J Intern Med.

[161] Song Y, Li Z, He T, Qu M, Jiang L, Li W, Shi X, Pan J, Zhang L, Wang Y (2019). M2 microglia-derived exosomes protect the mouse brain from ischemia-reperfusion injury via exosomal miR-124. Theranostics.

[162] Stahl SM (1998). Basic psychopharmacology of antidepressants, part 1: Antidepressants have seven distinct mechanisms of action. J Clin Psychiatry.

[163] Starobova H, Vetter I (2017). Pathophysiology of chemotherapy-induced peripheral neuropathy. Front Mol Neurosci.

[164] Sun J, Li Y-Z, Ding Y-H, Wang J, Geng J, Yang H, Ren J, Tang J-Y, Gao J (2014). Neuroprotective effects of gallic acid against hypoxia/reoxygenation-induced mitochondrial dysfunctions in vitro and cerebral ischemia/reperfusion injury in vivo. Brain Res.

[165] Techer D, Milla S, Fontaine P, Viot S, Thomas M (2015). Acute toxicity and sublethal effects of gallic and pelargonic acids on the zebrafish Danio rerio. Environ Sci Pollut Res.

[166] Teixeira AM, Trevizol F, Colpo G, Garcia SC, Charão M, Pereira RP, Fachinetto R, Rocha JB, Bürger ME (2008). Influence of chronic exercise on reserpine-induced oxidative stress in rats: behavioral and antioxidant evaluations. Pharmacol Biochem Behav.

[167] Tentori L, Graziani G (2009). Recent approaches to improve the antitumor efficacy of temozolomide. Curr Med Chem.

[168] Tieu K (2011). A guide to neurotoxic animal models of Parkinson’s disease. Cold Spring Harb Perspect Med.

[169] Tsoukalas D, Fragkiadaki P, Docea AO, Alegakis AK, Sarandi E, Thanasoula M, Spandidos DA, Tsatsakis A, Razgonova MP, Calina D (2019). Discovery of potent telomerase activators: unfolding new therapeutic and anti-aging perspectives. Mol Med Rep.

[170] Tsoukalas D, Fragkiadaki P, Docea AO, Alegakis AK, Sarandi E, Vakonaki E, Salataj E, Kouvidi E, Nikitovic D, Kovatsi L, Spandidos DA, Tsatsakis A, Calina D (2019). Association of nutraceutical supplements with longer telomere length. Int J Mol Med.

[171] Tsoukalas D, Zlatian O, Mitroi M, Renieri E, Tsatsakis A, Izotov BN, Burada F, Sosoi S, Burada E, Buga AM, Rogoveanu I, Docea AO, Calina D (2021). A novel nutraceutical formulation can improve motor activity and decrease the stress level in a murine model of middle-age animals. J Clin Med.

[172] Turrens JF (2003). Mitochondrial formation of reactive oxygen species. J Physiol.

[173] Variya BC, Bakrania AK, Madan P, Patel SS (2019). Acute and 28-days repeated dose sub-acute toxicity study of gallic acid in albino mice. Regul Toxicol Pharmacol.

[174] Vickers NJ (2017). Animal communication: when i’m calling you, will you answer too?. Curr Biol.

[175] Wahid M, Ali A, Saqib F, Aleem A, Bibi S, Afzal K, Ali A, Baig A, Khan SA, Bin Asad MHH (2020). Pharmacological exploration of traditional plants for the treatment of neurodegenerative disorders. Phytother Res.

[176] Wang YT, Lin HC, Zhao WZ, Huang HJ, Lo YL, Wang HT, Lin AM (2017). Acrolein acts as a neurotoxin in the nigrostriatal dopaminergic system of rat: involvement of α-synuclein aggregation and programmed cell death. Sci Rep.

[177] Weiss FU, Marques IJ, Woltering JM, Vlecken DH, Aghdassi A, Partecke LI, Heidecke CD, Lerch MM, Bagowski CP (2009). Retinoic acid receptor antagonists inhibit miR-10a expression and block metastatic behavior of pancreatic cancer. Gastroenterology.

[178] Wen L, Qu T-B, Zhai K, Ding J, Hai Y, Zhou J-L (2015). Gallic acid can play a chondroprotective role against AGE-induced osteoarthritis progression. J Orthop Sci.

[179] Wenk GL (2003). Neuropathologic changes in Alzheimer’s disease. J Clin Psychiatry.

[180] WFO. 2021. WFO The World Flora http://www.worldfloraonline.org/.

[181] Willis EF, Macdonald KPA, Nguyen QH, Garrido AL, Gillespie ER, Harley SBR, Bartlett PF, Schroder WA, Yates AG, Anthony DC, Rose-John S, Ruitenberg MJ, Vukovic J (2020). Repopulating microglia promote brain repair in an IL-6-dependent manner. Cell.

[182] Wojciechowski VV, Calina D, Tsarouhas K, Pivnik AV, Sergievich AA, Kodintsev VV, Filatova EA, Ozcagli E, Docea AO, Arsene AL, Gofita E, Tsitsimpikou C, Tsatsakis AM, Golokhvast KS (2017). A guide to acquired vitamin K coagulophathy diagnosis and treatment: the Russian perspective. Daru.

[183] Xie J, Shen Z, Anraku Y, Kataoka K, Chen X (2019). Nanomaterial-based blood-brain-barrier (BBB) crossing strategies. Biomaterials.

[184] Xu T, Kuang T, Du H, Li Q, Feng T, Zhang Y, Fan G (2020). Magnoflorine: a review of its pharmacology, pharmacokinetics and toxicity. Pharmacol Res.

[185] Yadav M, Jindal DK, Dhingra MS, Kumar A, Parle M, Dhingra S (2018). Protective effect of gallic acid in experimental model of ketamine-induced psychosis: possible behaviour, biochemical, neurochemical and cellular alterations. Inflammopharmacology.

[186] Yoshioka K, Kataoka T, Hayashi T, Hasegawa M, Ishi Y, Hibasami H (2000). Induction of apoptosis by gallic acid in human stomach cancer KATO III and colon adenocarcinoma COLO 205 cell lines. Oncol Rep.

[187] Yu M, Chen X, Liu J, Ma Q, Zhuo Z, Chen H, Zhou L, Yang S, Zheng L, Ning C (2019). Gallic acid disruption of Aβ1–42 aggregation rescues cognitive decline of APP/PS1 double transgenic mouse. Neurobiol Dis.

[188] Yu Z, Song F, Jin Y-C, Zhang W-M, Zhang Y, Liu E-J, Zhou D, Bi L-L, Yang Q, Li H (2018). Comparative pharmacokinetics of gallic acid after oral administration of gallic acid monohydrate in normal and isoproterenol-induced myocardial infarcted rats. Front Pharmacol.

[189] Zhang G, Zheng C, Huang B, Fei P (2020). Preparation of acylated pectin with gallic acid through enzymatic method and their emulsifying properties, antioxidation activities and antibacterial activities. Int J Biol Macromol.

[190] Zhang J-M, AN J (2007). Cytokines, inflammation and pain. Inte Anesthesiol Clin.

[191] Zhang Q, Yu Y-P, Ye Y-L, Zhang J-T, Zhang W-P, Wei E-Q (2011). Spatiotemporal properties of locomotor activity after administration of central nervous stimulants and sedatives in mice. Pharmacol Biochem Behav.

